# The first edentulous ceratosaur from South America

**DOI:** 10.1038/s41598-021-01312-4

**Published:** 2021-11-18

**Authors:** Geovane Alves de Souza, Marina Bento Soares, Luiz Carlos Weinschütz, Everton Wilner, Ricardo Tadeu Lopes, Olga Maria Oliveira de Araújo, Alexander Wilhelm Armin Kellner

**Affiliations:** 1 Programa de Pós-graduação em Zoologia (PPGZoo), Museu Nacional/Universidade Federal do Rio de Janeiro, Quinta da Boa Vista s/n, São Cristóvão, Rio de Janeiro, RJ 20940-040 Brazil; 2 Laboratório de Sistemática e Tafonomia de Vertebrados Fósseis (LAPUG), Departamento de Geologia e Paleontologia, Museu Nacional/Universidade Federal do Rio de Janeiro, Quinta da Boa Vista s/n, São Cristóvão, Rio de Janeiro, RJ 20940-040 Brazil; 3 Centro Paleontológico da Universidade do Contestado (CENPALEO), Universidade do Contestado, Av. Presidente Nereu Ramos, 1071, Jardim Moinho, Mafra, SC 89.306-076 Brazil; 4 Laboratório de Instrumentação Nuclear (LIN), Programa de Engenharia Nuclear/COPPE, Universidade Federal do Rio de Janeiro, Av. Horácio Macedo, Cidade Universitária, Rio de Janeiro, RJ 21941-450 Brazil

**Keywords:** Palaeontology, Taxonomy

## Abstract

The recognition of ontogenetic edentulism in the Jurassic noasaurid *Limusaurus inextricabilis* shed new light on the dietary diversity within Ceratosauria, a stem lineage of non-avian theropod dinosaurs known for peculiar craniomandibular adaptations. Until now, edentulism in Ceratosauria was exclusive to adult individuals of *Limusaurus*. Here, an exceptionally complete skeleton of a new toothless ceratosaur, *Berthasaura leopoldinae* gen. et sp. nov., is described from the Cretaceous aeolian sandstones of the Bauru Basin, Southern Brazil. The specimen resembles adult individuals of *Limusaurus* by the absence of teeth but based on the unfused condition of several elements (e.g., skull, vertebral column) it clearly represents an ontogenetically immature individual, indicating that it might never have had teeth. The phylogenetic analysis performed here has nested *Berthasaura leopoldinae* as an early-divergent Noasauridae, not closely related to *Limusaurus*. It represents the most complete non-avian theropod from the Brazilian Cretaceous and preserves the most complete noasaurid axial series known so far. Moreover, the new taxon exhibits many novel osteological features, uncommon in non-avian theropods, and unprecedented even among South American ceratosaurs. These include not only toothless jaws but also a premaxilla with cutting occlusal edge, and a slightly downturned rostral tip. This indicate that *B. leopoldinae* unlikely had the same diet as other ceratosaurs, most being regarded as carnivorous. As the ontogenetically more mature specimens of *Limusaurus*, *Berthasaura* might have been herbivorous or at least omnivorous, corroborating with an early evolutionary divergence of noasaurids from the ceratosaurian bauplan by disparate feeding modes.

## Introduction

Ceratosauria represents one of the most widespread and diverse clade of extinct theropods^1,2^. Currently, three main lineages are recognized within Ceratosauria: Ceratosauridae, Abelisauridae and Noasauridae^3–5^. In terms of morphology, mid- to large-sized members of the Abelisauridae and Ceratosauridae are relatively better-known than the gracile small-bodied noasaurids. Most noasaurids consist primarily of fragmentary specimens, with few exceptionally well-preserved taxa restricted to the Malagasy species *Masiakasaurus knopfleri*^6^, the Tanzanian *Elaphrosaurus bambergi*^5^, and *Limusaurus inextricabilis*^4,7^ from China. Despite the fragmentary nature of the noasaurid fossil record, it revealed some osteological features uncommon among non-avian theropods, incluing procumbent and heterodont lower dentition^6,8,9^ or toothless rostra likely covered by rhamphothecae^4^. These ceratosaurs indicate a complex evolution of feeding linked to the origin and diversification of the Noasauridae.

Not surprisingly, a robust understanding of the evolutionary history and distribution of the morphoanatomical traits of noasaurids, and their meaning within a broader ceratosaur and theropod evolutionary context, is still in progress. As early as in the Late Jurassic, noasaurids present a highly derived combination of characters^4,7^. The scarcity of knowledge regarding early diverging species and the temporal and ‘morphological’ gaps between Late Jurassic and Late Cretaceous noasaurids, strongly limit our understanding of the evolution of the group^5^. Recently, a paleontological site named *Cemitério dos Pterossauros* Quarry (Pterosaur Graveyard)^10^ from the Lower Cretaceous Goio Êre Formation (Bauru Basin), which crops out at the Northwestern Paraná State, Southern Brazil, revealed several theropod remains^11,12^. Here, we describe a new edentulous noasaurid yielded from this site which is also the first toothless non-avian theropod known from Brazil.

### Institucional abbreviations

MN—Museu Nacional, Universidade Federal do Rio de Janeiro, Rio de Janeiro, Brazil; IVPP—Institute of Vertebrate Paleontology and Paleoanthropology, Beijing, China; MPCO.V—Museu de Paleontologia de Cruzeiro do Oeste, Cruzeiro do Oeste, Brazil; UA—Université d’Antananarivo, Antananarivo, Madagascar; USNM—National Museum of Natural History, Washington DC.

## Results

### Systematic paleontology

- Dinosauria Owen, 1842
- Theropoda Marsh, 1881
- Abelisauroidea (Bonaparte and Novas, 1985) sensu Wilson et al.^13^
- Noasauridae Bonaparte & Powell, 1980 sensu Wilson et al.^13^
- *Berthasaura leopoldinae* gen. et sp. nov.

#### Etymology

The generic name honors the researcher Bertha Maria Júlia Lutz (1894–1976) for her scientific contribution and social activity particularly regarding woman rights in Brazil, combined with *saura*, feminine of *saurus* from the Greek for lizard. The specific epithet *leopoldinae* honors the first Brazilian empress, Maria Leopoldina (1797–1826), for her fundamental role in the independency of Brazil that next year (2022) will complete two centuries; and the samba school Imperatriz Leopoldinense, that in the 2018 carnival developed the theme "A Royal Night at the Museu Nacional" (Uma noite real no Museu Nacional) in commemoration of the bicentenary of the museum. This was before the large fire of September 2018^14^.

#### Holotype

**Figure 1:**
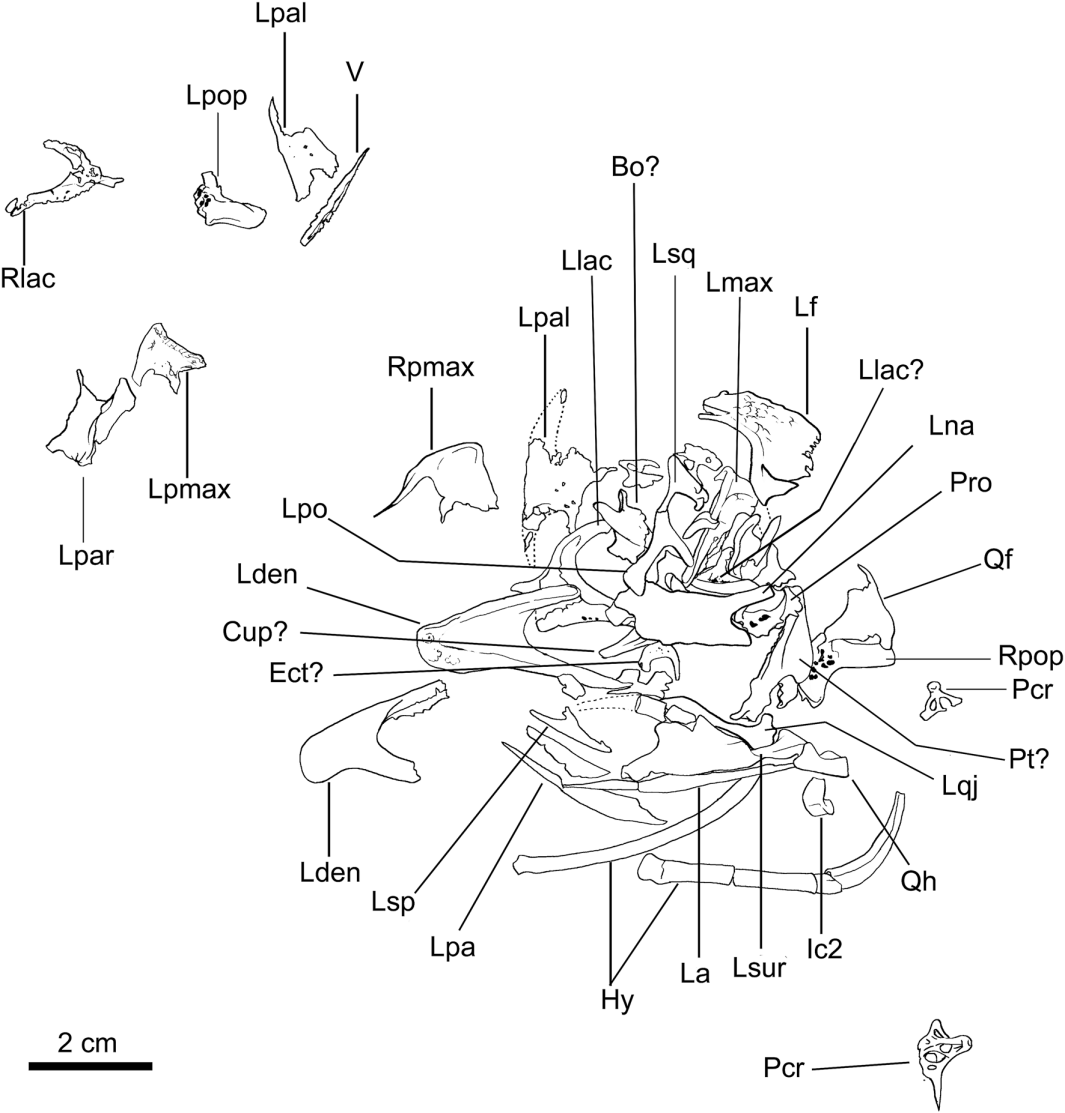
*Berthasaura leopoldinae* gen. et sp. nov*.* holotype (MN 7821-V), line drawing of the skull and mandible. Abbreviations: Bo? basioccipital fragment; Cup?, cutriform process; Ect? ectoperygoid; Hy, hyoids; Ic2, intercentrum 2; La, left angular; Lden, left dentary; Lf, left frontal; Llac, left lacrimal; Llac?, left lacrimal fragment; Lmax, left maxilla; Lna, left nasal; Lpa, left prearticular; Lpal, left palatine; Lpo, left postorbital; Lpar, left parietal; Lpmax, left premaxilla; Lpop, left paraoccipital process; Lqj, left quadratojugal; Lsp, left splenial; Lsq, left squamosal; Lsur, left surangular; Pcr, proximal fragment of cervical rib; Pro, prootic fragment; Pt?, pterygoid; Qf, quadratic flange (pterygoid flange of the quadrate); Qh, quadratic head; Rden, right dentary; Rlac, right lacrimal; Rpop, right paraoccipital process; Rpmax, right premaxilla. Line drawing by G.A.S.

**Figure 2:**
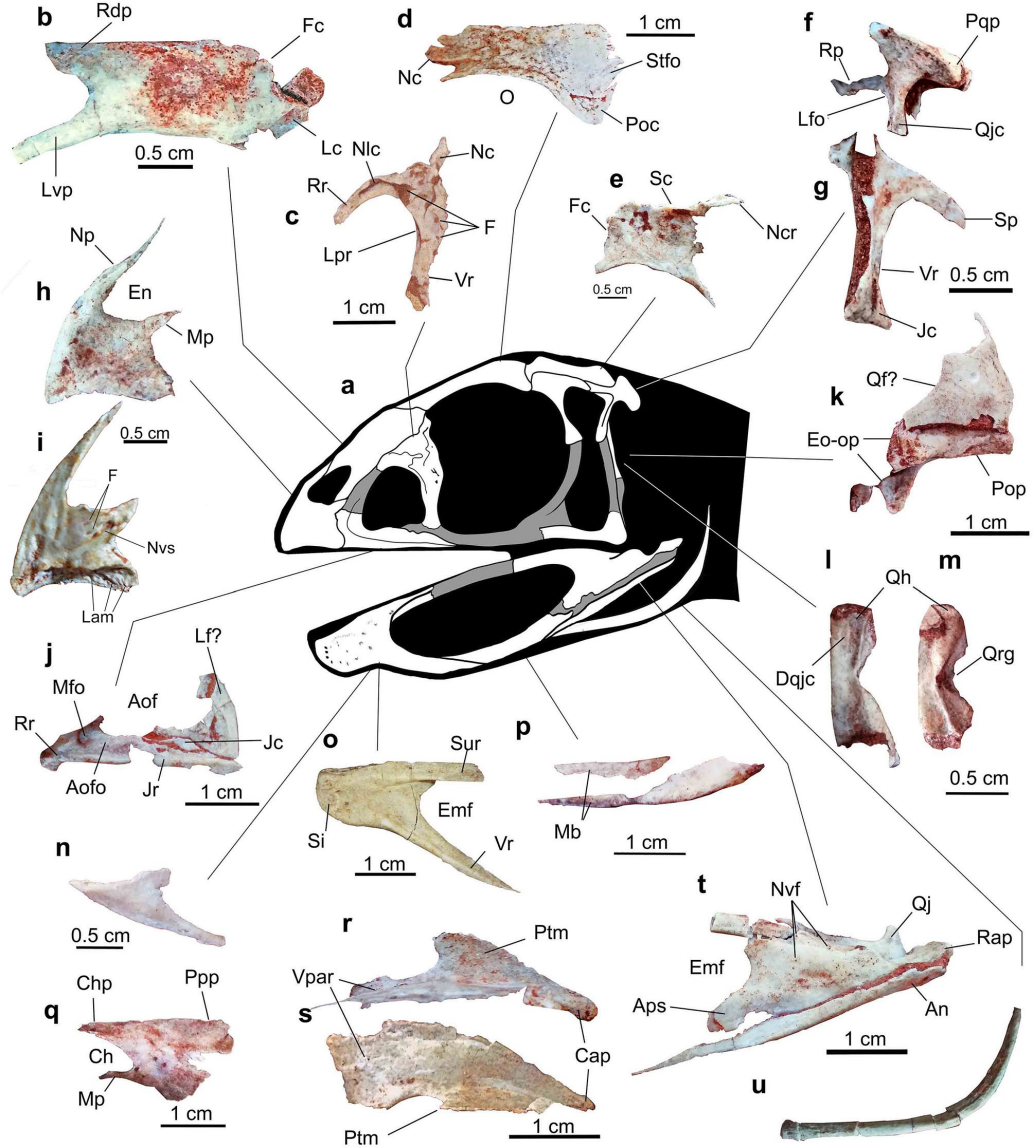
*Berthasaura leopoldinae* gen. et sp. nov*.* holotype (MN 7821-V), interpretative line drawing of the skull and photographs of cranial bones. (**a**) Interpretative reconstruction of the skull. (**b**) Left nasal in dorsolateral view. (**c**) Right lacrimal in lateral view. (**d**) Left frontal in dorsal view. (**e**) Left parietal in dorsal view. (**f**) Left squamosal in lateral view. (**g**) Left postorbital in lateral view. (**h**) Left premaxilla in lateral view. (**i**) Right premaxilla in medial view. (**j**) Left maxilla in lateral view. (**k**) Left paraoccipital in caudal view and left (?) quadratic flange. (**l, m**) Quadratic head in lateral and caudolateral views, respectively. (**n**) Left splenial in lateral view. (**o**) Right dentary in lingual view. (**p**) (p) Left prearticular in lateral view. (**q**) Left palatine in dorsal view. (**r**, **s**) Left pterygoid in lateral and dorsal views, respectively. (**t**) Left surangular and angular in lateral view. (**u**) Left ceratohyal in lateral view. Abbreviations: An, Angular; Aof, Antrorbital fenestra; Aofo, Antorbital fossa; Aps, Angular process of surangular; Ch, Choana; Chp, Choanal process; Cap, Caudal process; Cvp, Caudoventral process; Dqjc, Dorsal quadratojugal contact; Emf, External maxillary fenestra; En, External naris; Eo-op, Exoccipital-ophistotic; F, Foramina; Fc, Frontal contact; Jc, Jugal contact; Jr, Jugal ramus; Lam, lamellae; Lc, Lacrimal contact; Lf, Lacrimal fragment? Lfo, Lateral fossa; Lpr, Lacrimal pneumatic reces; Lvp, Lateroventral process of nasal; Mb, Medial body of prearticular; Mfo, Maxillary fossa; Mp, Maxillary process; Nc, Nasal contact; Ncr, Nuchal crest; Nlc, Nasolacrimal canal; Np, Narial process; Nvf, Neurovascular foramina; Nvs, Neurovascular sulcus; O, Orbit; Poc, Postorbital contact; Pop, Paraoccipital process; Ppp, Pterygoid process of palatine; Pqp, Postquadratic process; Ptm, Pterygoid medial process; Qf?, Quadrate flange (pterygoid flange of the quadrate); Qh, Quadratic head; Qj, Quadratojugal; Qjc, Quadratojugal contact; Qrg, Quadrate ridge groove; Rap, Retroarticular process; Rdp, Rostrodosal process of nasal; Rr, Rostral ramus; Sc, Sagittal crest; Si, Symphyseal region; Sp, Squamosal process; Stfo, Supratemporal fossa; Sur, Surangular ramus; Vpar, Vomeropalatine process of pterygoid; Vr, Ventral ramus. Line drawing by G.A.S.

**Figure 3:**
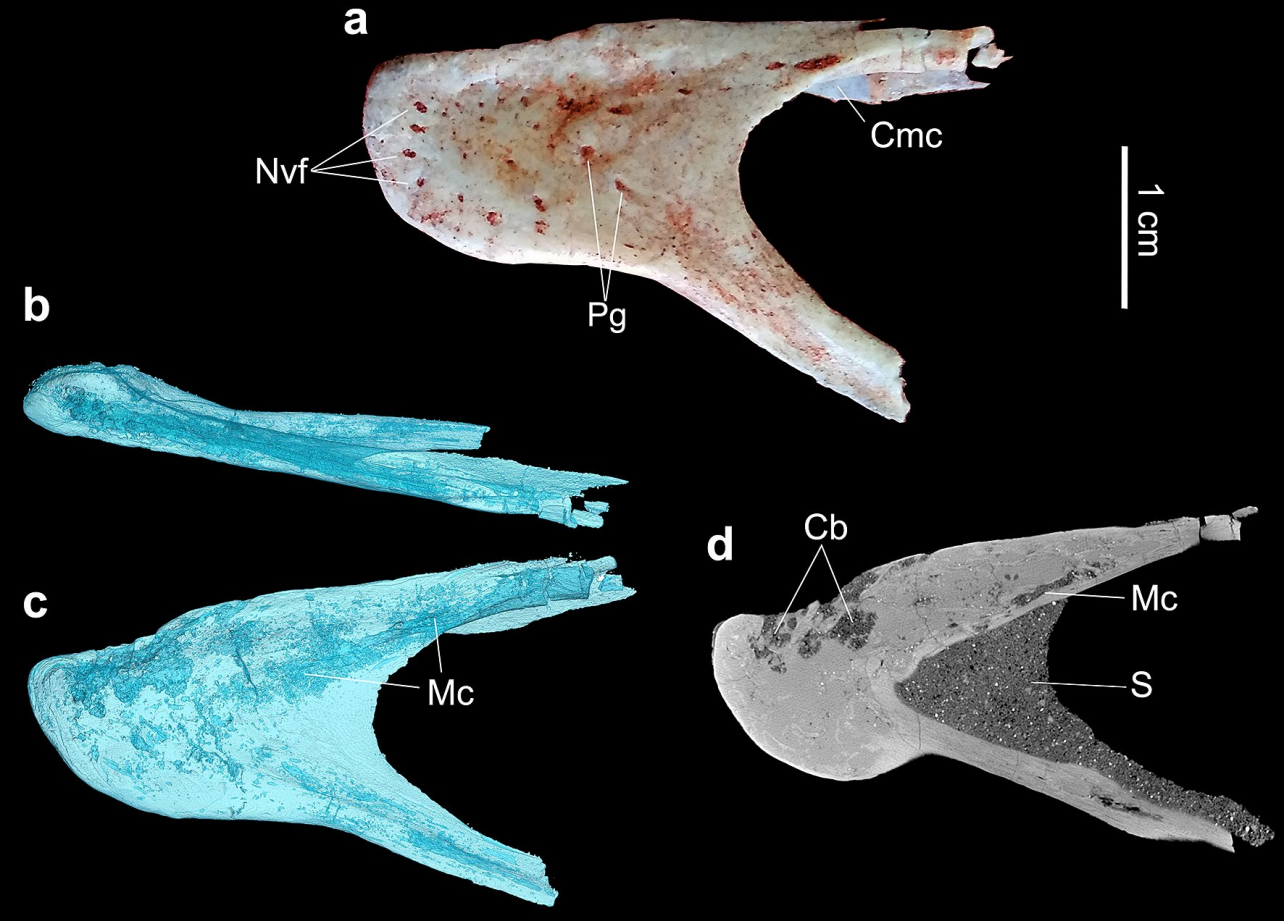
Photograph and inner structure of the left dentary. (**a**) Photograph of the dentary in lateral view. (**b**, **c**) µCT-scan of the dentary in occlusal and lateral views, respectively. (**d**) Slice showing the cavities within the trabecular bone connected with the outer bone surface by foramina, in sagittal view. Abbreviations: Cb, Cavities of trabecular bone; Cmc, Caudal open of Meckelian canal; Mc, Meckelian canal; Nvf, neurovascular foramina; Pg, Pits and grooves; S, sediment.

**Figure 4:**
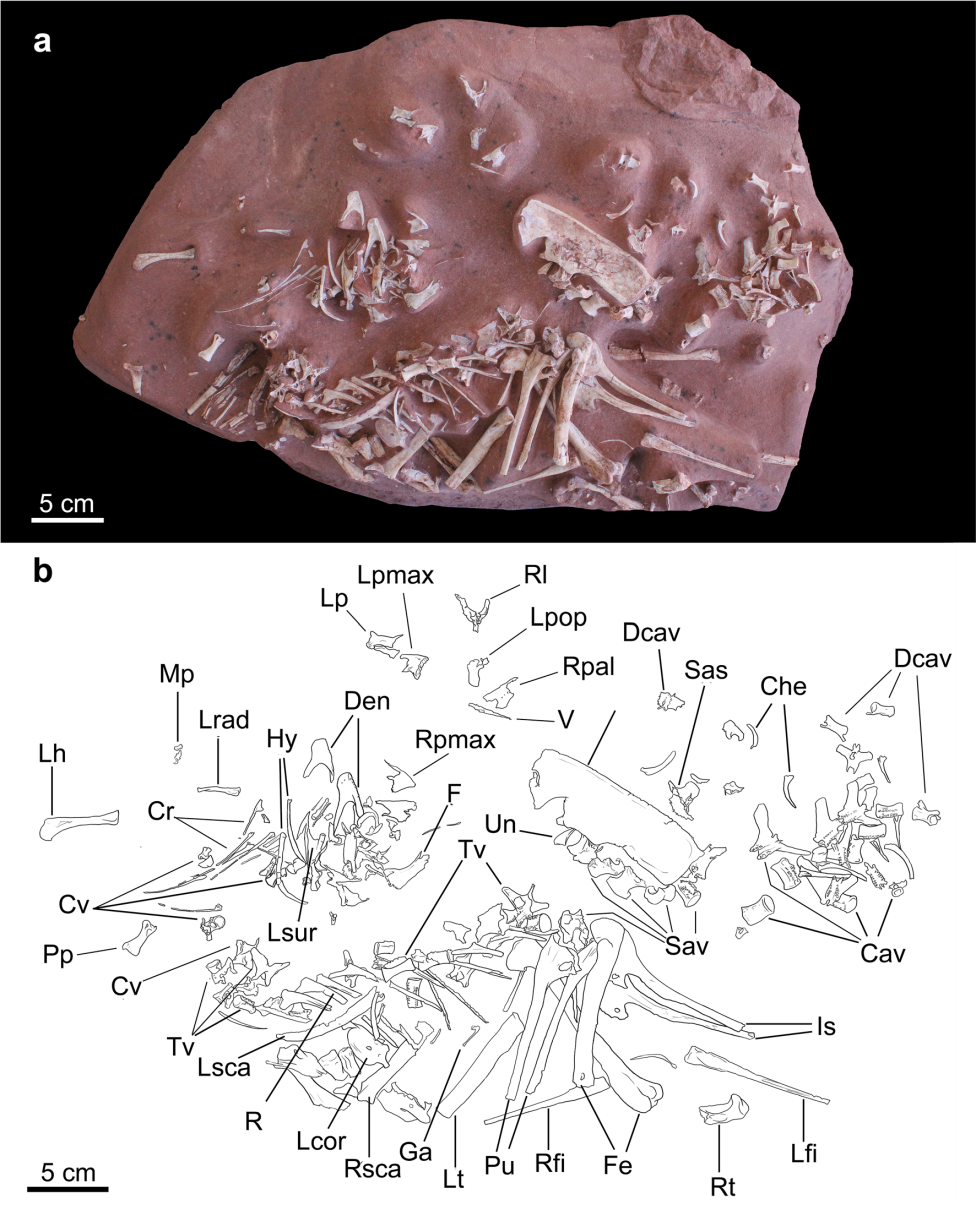
*Berthasaura leopoldinae* gen. et sp. nov*.*, holotype (MN 7821-V), nearly complete skeleton. (**a**) Photograph. (**b**) Respective line drawing. Abbreviations: Cav, Caudal vertebrae; Che, Chevrons; Cr, Cervical ribs; Cv, Cervical vertebrae; Dcav, Distal caudal vertebrae; Den, Dentaries; F, Frontal; Fe, Femora; Ga, Gastral elements; Hy, Hyoids; Is, Ischia; Lcor, Left coracoid; Lfi, Left fibula; Lh, Left humerus; Lp, Left parietal; Lpmax, Left premaxilla; Lrad, Left radius (ulna hidden); Lsca, Left scapula; Lsur, Left surangular; Lt, Left tibia; Mp, Manual phalanges; Pp, left pedal phalanx III-1; Pu, Pubes; R, Trunk ribs; Rfi, Right fibula; Rl, Right lacrimal; Rpal, Right palatine; Rpmax, Right premaxilla; Rsca, Right scapula; Rt, Right tibia fragment; Sas, Sacral neural spine; Sav, Sacral vertebrae; Tv, Trunk vertebrae; V, Vomeropterygoid process fragment. Line drawing by G.A.S.

**Figure 5:**
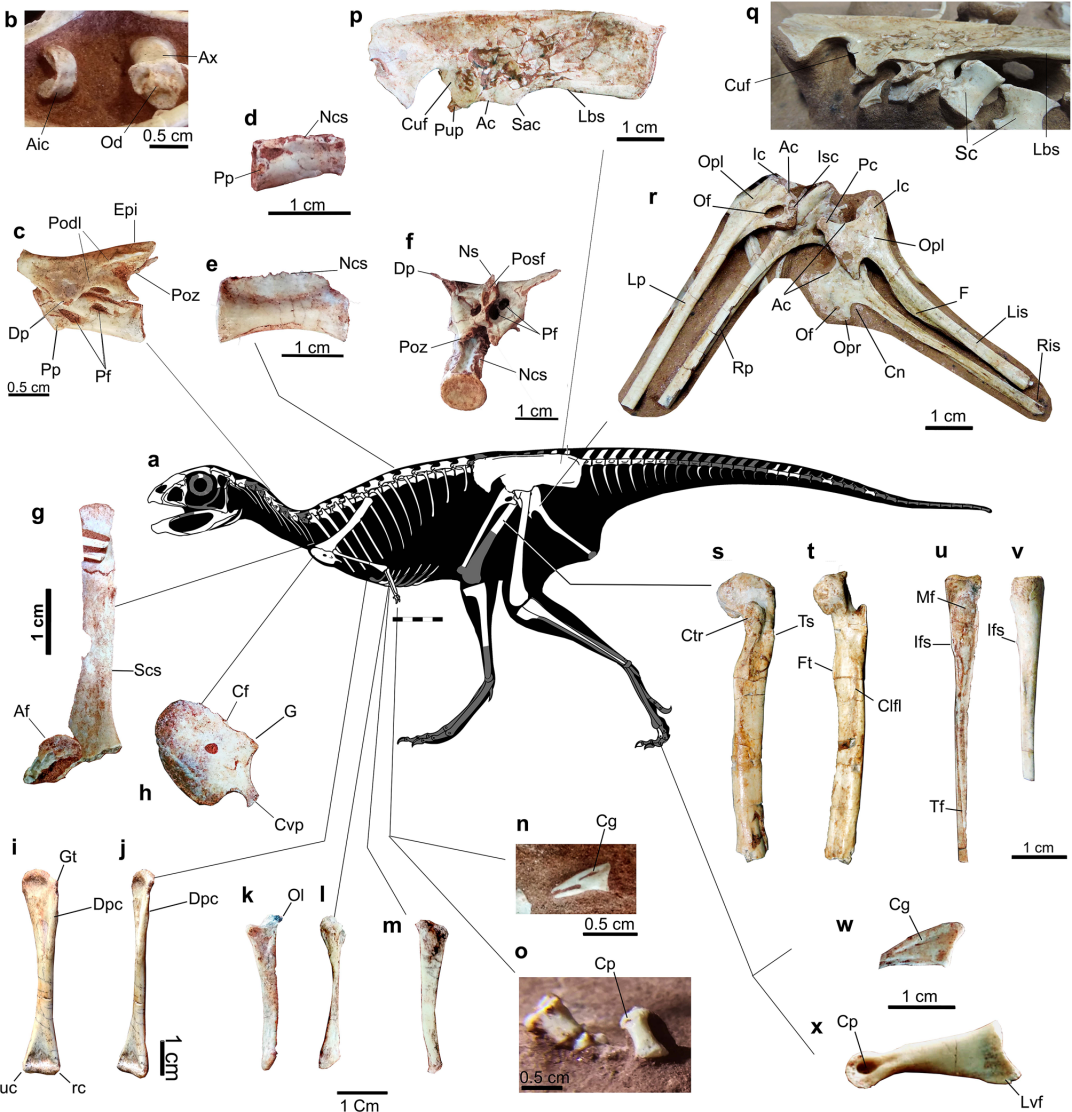
Interpretative line drawing of *Berthasaura leopoldinae* gen. et sp. nov*.*, holotype (MN 7821-V), and photographs. (**a**) Line drawing reconstruction; (**b**) Intercetrum and axis in cranioventral views; (**c**) 4th cervical vertebrae in lateral view; (**d**) First trunk centrum in lateral view; (**e**) 8th trunk centrum in lateral view; (**f**) last sacral vertebrae in caudal view; (**g**) right scapula in medial view; (**h**) left coracoid in lateral view; (**i**, **j**) left humerus in cranial and lateral views, respectively; (**k**, **l**) left ulna in lateral and cranial views, respectively; (**m**) left radius in cranial view; (**n**) manual ungual in lateral view; (**o**) manual phalanges II (in dorsal view) and III (in lateral view); (**p**) left ilium in lateral view; (**q**) left ilium in ventral view; (**r**) pubes and ischia; (**s**, **t**) left femur in cranial and medial views, respectively; (**u**) left fibula in medial view; (**v**) right fibula in lateral view; (**w**) pedal ungual in lateral view; (**x**) left pedal phalanx III-1 in lateral view. Abbreviations: Ac, Acetabular margin; Af, Acromion fragment; Aic, Axial intercentrum; Cf, Coracoid foramen; Cg, Colateral vascular groove; Clfl, Fossa for attachment of *M. Caudofemoralis longus*; Cn, Caudal notch; Cp, Colateral pit; Ctr, Cranial trochanter; Cuf, Cuppedicus fossa; Cvp, Caudoventral process; Dp, Diapophysis; Dpc, Deltopectoral crest; Epi, Epipophysis; F, Flange; Ft, Fourth trochanter; G, Glenoid; Gt, Greater trochanter; Ic, Iliac contact; Ifs, scar for *M. iliofibularis*; Isc, Ischial contact; Lbs, Lateral brevis shelf; Lis, Left ischium; Lp, Left pubis; Lvf, Lateroventral flange; Mf, Medial fossa; Clfl, Fossa for *M. caudofemoralis longus*; Ncs, Neurocentral suture; Ns, Neural spine; Od, Odontoid process; Ax, Axial centrum; Of, Obturator foramen; Ol, Oleacron; Opl, Obturator plate; Opr, Obturator process; Pc, Pubic contact; Pf, Pneumatic foramina; Podl, Postzygodiapophyseal lamina; Posf, Post spinal fossa; Poz, Postzygapophysis; Pp, Parapophysis; Pup, Pubic peduncle; Rc, Radial condyle; Ris, Right ischium; Rp, Right pubis; Sac, Supraacetabular crest; Scs, Scapular shaft; Ts, Trochanteric shelf; Uc, Ulnar condyle. Line drawing by G.A.S.

MN 7821-V comprises a nearly complete disarticulated skeleton, including a partial skull and lower jaw (Figs. 1, 2, 3, 4, and 5).

#### Horizon and locality

*Cemitério dos Pterossauros* Quarry^10^, Goio Erê Formation, Early Cretaceous (Aptian-Albian), Caiuá Group, Bauru Basin^15^. The fossils were collected in layers that crop out in a rural road at Cruzeiro do Oeste Municipality, Paraná State, Southern Brazil.

#### Diagnosis

Noasaurid ceratosaur with the following combination of characters that distinguishes it from other members of the clade (autapomorphies are marked with an asterisk): edentulous rostrum (premaxilla, maxilla and dentary); lamellae on the medial surface of the premaxilla*; short dentaries with the length rostral to the mandibular fenestra about 1.5 times the height of the dentary*; protuberance projecting from the caudoventral end of the premaxillary buccal rim*; non-bifurcated rostral end of splenial*; postzygodiapophyseal lamina in mid-cervical vertebrae divided in two parts and reduced to low ridges; maximal length of metacarpus less than 15% of the length of humerus; iliac blade mediolaterally flattened; medial brevis shelf strongly reduced*; deep notch on the caudal margin of the ischial process producing an eminent and caudally-oriented prong*; rounded medial femoral epicondyle.

### Ontogenetic assessment

Establishing the ontogenetic stage in any fossil vertebrate can be quite challenging due to the lack of complete and sometimes comparable ontogenetic sequences^16–18^. The multiple specimens referred to *Limusaurus inextricabilis* enable the establishment of six ontogenetic stages based mainly on size and osteohistological sections and an attempt to assess variation in anatomy to ontogeny^4^. This may provide a parameter to infer the degree of maturity of *Berthasaura leopoldinae*, with the caveat that it is not sure at the time being if these noasaurid species share the same ontogenetic development.

From the 73 anatomical features listed by Wang et al.^4^, they only refer to the fusion of bones in three: fusion of astragalus and calcaneum to each other and to the distal end of tibia, posterior cervical ribs fused with the respective centrum, and fusion of the frontal with the parietal. The holotype of *Berthasaura leopoldinae* lacks the distal end of tibia + astragalus and calcaneum, but in any case, the fusion of these elements has proven to be of little ontogenetic information for *Limusaurus*, since in all stages these elements are unfused and some variation occurs at stage 5^4^. Furthermore, Souza et al.^12^ showed osteohistological sections of a tibia unfused to the proximal tarsal elements from a noasaurid collected in the same locality where *Berthasaura* was found, with an external fundamental system, indicating that the relation between the fusion of these elements with ontogeny is not clear. In *Limusaurus inextricabilis*, fused cervical ribs with the respective centrum in posterior neck vertebrae are found in ontogenetic stage 5^4^. Although a complete neck is not available for *Berthasaura leopoldinae*, the preserved posterior cervical elements lack fused cervical ribs, suggesting that the MN 7821-V is younger than ontogenetic stage 5.

Still regarding *Limusaurus inextricabilis*, parietal and frontal are not fused in ontogenetic stages 1 and 2, fused in ontogenetic stage 4, and unknown in ontogenetic stage 3^4^. These bones are unfused in the holotype of *Berthasaura leopoldinae*, indicating that MN 7821-V must be younger than ontogenetic stage 4, perhaps even than ontogenetic stage 3.

It should be noted that in MN 7821-V all preserved cervical elements show open sutures between the neural arch and the centrum, and all dorsal vertebrae have the centrum and neural arches unfused. Furthermore, all cranial elements are unfused except for the surangular and the articular which are tightly connected without an apparent open suture. Other potential ontogenetic features used in *Limusaurus inextricabilis* by Wang et al.^4^ is the presence of the posterior process of the quadratojugal, the configuration of the anterior portion of the dentary, the presence of epipophyses on anterior cervical vertebrae, the degree of development of the parapophyseal pedicles, development and shape of the coracoid tubercle, and the proportion and shape of several long bones. Although the conditions are known for ontogenetic stages 2 and 4, they remain unknown for ontogenetic stage 3. In all these features, the holotype of *Berthasaura leopoldinae* shows the condition equivalent to an ontogenetic stage 4 and differ from the ontogenetic stage 2.

Other features used to infer ontogenetic maturity in reptiles are the degree of ossification of the articulations in long elements and the texture of the bones^16,18^. In MN 7821-V all preserved long bones (e.g., humerus, radius, ulna, femur, tibia) show the articulations well developed and the bone surface is well ossified, lacking the characteristic pits found in ontogenetic immature individuals^16,19,20^. Based on the available information, it can be concluded that the holotype of *Berthasaura leopoldinae* was neither an early juvenile nor an adult individual. It most likely was a young sub-adult at time of death, comparable to ontogenetic stages 3 or 4 of *Limusaurus inextricabilis*.

### Description and comparisons

*Berthasaura leopoldinae* n. gen. et sp. represents the most complete known noasaurid species from Brazil (Figs. 1, 2, 3, 4, and 5). Based on comparisons with more complete ceratosaurs, the specimen MN 7821-V represents a small theropod, with an estimated body length not exceeding 1 m. It was smaller than *Vespersaurus*^11^, *Velocisaurus unicus*^21,22^, adult specimens of *Limusaurus*^4^, and the smallest associated specimen (FMNH PR 2485) of *Masiakasaurus*^9^. The entire list of measurements (in mm) can be found as Supplementary Table S1.

The premaxilla is transversally laminar. When articulated, in ventral view, the premaxillae are ‘V-shaped’. The rostral tip bows medially and ventrally. In transversal section, the premaxilla tapers ventrally, featuring a thin buccal cutting border, resembling specialized herbivorous dinosaurs such as the therizinosaur *Erlikosaurus*^23^. The buccal border shows a series of lamellae that are vertically to obliquely oriented relative to the buccal margin (Fig. 2i), resembling the lamellae of the ornithomimosaurian *Gallimimus*, hadrosaurid ornithischians (e.g., *Edmontosaurus*), and herbivorous chelonians^24^. The lateral surface of the premaxilla lacks apparent neurovascular foramina (Fig. 2h). The narial process is narrow, with a dorsoventrally flattened distal half. Ventral to the narial process, a smaller maxillary process protrudes caudally on the mid-height of the premaxilla, participating in the rostroventral margin of the naris. Ventrally, a third expansion protrudes from the premaxillary body towards the maxilla. This expansion caudally exceeds the maxillary process.

The rostral ramus of maxilla protrudes from the main body of the element. It runs rostrally while bowing medially (together with the rostralmost portion of the maxillary main body), suggesting that the premaxilla partially overhangs it. This medial curvature features a gentle arc that provides a half ‘U-shape’ to the rostralmost portion of the maxilla in ventral view and would form the roof of the palate, meeting its counterpart on the right maxilla. The buccal surface of the jugal ramus is flat and continuous with the ventral margin of the rostral ramus and maxillary main body, providing a straight and horizontal outline to the maxilla in lateral view, similar to other noasaurids^6,7,25^. A pocket lies on the lateral surface of the jugal ramus of the maxilla that receives the rostral process of the jugal (Fig. 2j). The antorbital fossa covers most of the lateral surface of the maxilla. A shallow maxillary fossa lies on the rostroventral corner of the antorbital fossa, similar to coelophysoids^26^, *Ceratosaurus*^27^, and noasaurids^6,8^. As in the premaxilla, teeth are absent.

The lacrimals are fairly well-preserved (Fig. 2c) but lack the distal part of the descending process, not allowing to establish how this element contacts the jugal or the maxilla, an important feature within theropod phylogeny^28,29^. In the lateral view, the lacrimal is ‘C-shaped’ contrasting with the ‘L-shaped’ and the ‘T-shaped’ condition of *Limusaurus*^4,7^ and abelisaurids^30,31^, respectively. A deep and non-septate pneumatic recess lies at the antorbital margin. The recess of this bone separates the lateral and medial walls, with the former hiding the latter in lateral view. A pneumatic foramen is visible on the lateral wall of the lacrimal recess, while smaller neurovascular foramina lie at the lateroventral corner of this bone.

Only the left nasal is preserved and was not fused with its right counterpart. In contrast to many abelisaurids^19–21,30,31^ but similar to *Limusaurus*^4,7^, the external nasal surface is smooth and lacks both rugosities and foramina (Fig. 2b). This bone in the Chinese taxon is shorter and lacks an extended lateroventral process.

The frontal shows a smooth external surface and is rostrocaudally longer than lateromedially wide. A deep pit surrounded by a fossa lies at the rostrolateral corner, differing from the ‘socket-like’ slot of *Masiakasaurus*^9^. A near triangular expansion (= postorbital process) protrudes towards the postorbital on the caudolateral corner. The supratemporal fossa is narrow and angular.

As in *Limusaurus*^4,7^, the parietal lacks a rugose external surface which is present in several abelisaurids^30–32^. A relatively low sagittal crest extends along the medial margin. Caudally, an eminence projects dorsocaudally approaching an angle of about 45° relative to the main rostrocaudal axis, forming part of the nuchal crest.

A gracile and triradiated bone in close association with the squamosal is tentatively identified as the left postorbital (Figs. 1 and 2g). In lateral view, it has a biconcave outline, with the presumably caudal margin approaching a more acute angle than the rostral margin (Fig. 2g). It is ‘T-shaped’ as in several theropods^33,34^, rather than the ‘C-shaped’ condition of *Masiakasaurus*^9^ and abelisaurids^19,20,30–32^. The ventralmost end of the ventral ramus expands into a laminar fan-like contact for the jugal.

The squamosal exhibits a tetraradiate condition (Fig. 2f), as observed in *Limusaurus*^7^, contrasting with the triradiate squamosal of many abelisaurids^19,20^. Its caudal margin forms the postquadratic process that is more robust and caudally projected than those of *Ceratosaurus*^27^, *Majungasaurus*^31^, *Carnotaurus*^30^, and *Rajasaurus*^13^, but lesser than *Abelisaurus*, and similar to *Skorpiovenator*^32,35^. The quadratojugal process protrudes ventrally, approaching an angle of 90° with the rostrodorsal process for the postorbital. Its ventralmost end is missing. A lateral fossa lies along the lateral corner between the rostrodorsal and quadratojugal processes, comprising the dorsocaudal margin of the infratemporal fossa.

The quadratojugal exhibits the typical “L-shape” of many theropods including *Limusaurus*^4^. The rostral portion has a low and smooth ridge protruding dorsally. It provides a gentle, undulating outline to the jugal ramus, in lateral view. The caudal corner of the quadratojugal bears a caudoventral process. The squamosal process is broken near its base, but it approaches 90° relative to the jugal ramus.

The palatal region of noasaurids has not been described. The palatine of *Berthasaura* is tetraradiate, thin and rostrocaudally elongated, differing from the rostrocaudally shortened palatines of *Carnotaurus* and *Majungasaurus*. A huge laminar and crested bone was tentatively identified as the left pterygoid (Fig. 2r, s). The element has a concave surface facing ventrally. The vomeropterygoid process is lateromedially wide and the outline is continuum with the main pterygoid body, as in *Carnotaurus*, but lacks the dorsoventrally deep aspect of this abelisaurid^30^. The caudal end features a downturned process, as in *Ceratosaurus*^27^. The quadratic head is the only portion of the left quadrate that is definitely preserved (Fig. 2l, m). An ‘aliform’, plate-like and fragmented bone, separate from the quadrate, was interpreted as the pterygoid flange of the left quadrate (Fig. 2k). The caudal surface of quadratic head is deeply concave. Two ‘lip-like’ protuberances raise from the caudal surface of the quadratic head, surrounding the caudal concavity medially and laterally. A groove deeply incises on the mid-height of this surface, interrupting these protuberances, featuring the quadratic ridge groove.

The dentary is toothless, what was confirmed by computed microtomography (μCT) scanning (Figs. 2o and 3). It has a downturned symphysis with thickened rostral end relative to the rest of bone, as in the ontogenetic stage 4 of *Limusaurus* (IVPP V 15923)^4^. The dentary is rostrocaudally shorter and dorsoventrally taller than that of *Limusaurus*. In dorsal view, each dentary ramus is straight, providing a V-shape to the mandible when articulated, similar to *Masiakasaurus*^6^. Both surangular and ventral rami bound an exceptionally larger external mandibular fenestra (EMF). The buccal border is straight and horizontal, caudal to symphyseal inflection, as in *Limusaurus*. It shows simplification of the occlusal surface, with a smooth and longitudinal groove lying on it. The µCT scan data showed that alveolar vestiges are absent and/or modified to trabecular cavities inside the dentary of *Berthasaura leopoldinae* (Fig. 3d). Labially, the neurovascular foramina are not displayed in a row, instead, pits and grooves occur dispersed on the labial surface (Fig. 3a) of the dentary and face caudally, as those in *Confuciusornis* and other edentulous theropods, suggesting the presence of a rhamphotheca^36–38^.

A small plate-like triangular fragment was interpreted as the left splenial (Fig. 2h). As with the Meckelian fossa of the dentary, the splenial is rostrocaudally short. A small recess lies longitudinally at its ventral margin, likely to articulate with the ventral rim of the main body of the dentary. The surangular is broken at its rostral portion unrevealing the articulation with the dentary. The rostral portion of the surangular is shallow in lateral view, similar to *Limusaurus*^4^ and coelophysoids^26^. The lateral ridge present in many theropods is absent. The glenoid is relatively wide and its concavity faces dorsally instead of craniomedially, as in *Masiakasaurus*^9^. An elongated retroarticular process projects caudally and dorsally. This process is proportionally longer than all other ceratosaurs and most non-avian theropods. The angular process of the surangular inserts dorsally at the rostral third of the angular.

The angular projects rostrally to the surangular and both form the caudoventral border of the EMF. Rostrally, the angular tapers to a splint-like bone and ends caudally at the caudalmost level of the retroarticular process.

The postcranial axial skeleton of *Berthasaura leopoldinae* (Figs. 4 and 5) preserves atlantal and axial intercentra, axial centrum, four postaxial cervical vertebrae (mid- to caudal), 11 trunk vertebrae, five unfused sacrals, and 16 vertebrae from different parts of the tail (proximal, mid-distal and distal). This count ranges within the expected for Ceratosauria based on the most complete specimens of *Majungasaurus*^39^ and *Masiakasaurus*^9^.

The atlas intercentrum is semilunar in cranial/caudal view, with its concave surface facing dorsally. The axis intercentrum consists of two reniform portions laterally positioned to each other (Fig. 5b). These portions are confluent ventrally until fused in a mid-line in the ventral surface, providing a “v-shape” in cranial view. The dorsal groove of the ‘V’ articulates with the odontoid process on axis. The cranial portion of the axial centrum is in the same level of the caudal portion, differing from the axis of *Vespersaurus*^11^, which exhibits a more sigmoidal fashion in lateral view. A “D-shaped” odontoid projects at the dorsal half of the cranial articular facet. A hypertrophied rim marks the limit between the cranial articular facet and the axial centrum. The lateral walls lack a pneumatic foramen. The axial neural spine is hidden by the matrix and cranial bones.

The mid-cervical vertebra (probably 4th or 5th) of *Berthasaura* possess two pneumatic foramina perforating pleurocoels (Fig. 5c), which is not observed in the caudalmost cervical vertebrae. In all preserved post-axial cervical vertebrae, the cranial articular facets are flattened, whereas the caudal facets are concave. In the mid-cervical vertebrae, a bony lamina subdivides the dorsal surface of the centrodiapophyseal fossa in two small fossae: a wider one close to the centrum, and a smaller fossa, craniolateral to the former, and placed on the abaxial surface of diapophysis apex. At least at the cranialmost post-axial vertebra, a low postzygodiapophyseal lamina is divided in parts, as in *Elaphrosaurus*. The epipophysis projects caudally beyond to the postzygapophysis in the mid-cervical vertebra, as in the fourth cervical of *Masiakasaurus*^9^, but differing from *Vespersaurus*, which has epipophyses that do not exceed the articular facet of the postzygapophyses. The cervical diapophyses do not exceed ventrally the mid-point level of the centra in *Berthasaura leopoldinae*, as in the referred cervicals of *Vespersaurus* (MPCO.V 0017, MPCO.V 0067)^11^ and the cervicals of *Masiakasaurus*^9^, but differing from the isolated cervical vertebrae from Adamantina Formation noasaurid (DGM 929-R)^40^. The neural spine is preserved in a single cervical vertebra, probably belonging to the caudal half of the neck. This neural spine is extremely low, not exceeding 1/3 of the neural arch height.

All trunk centra are externally apneumatic, as in *Masiakasaurus* and other ceratosaurs. The centra, however, show an increase in craniocaudal length toward the caudal half of the trunk (Fig. 5d, e), differing from the uniform condition of *Masiakasaurus*. The centra length in the cranial half ranges between 1.2 and 1.5 cm, whereas in the caudalmost half of the trunk exhibit about 1.9 cm length (Supplementary Table S1). The mid-length of centra become highly constricted toward the caudal half of the series (the ratio between cranial or caudal ends width relative to the mid-centrum width is 2.3–2.6), as in *Elaphrosaurus*^5^*.*

The cranial ends of the centra are displaced ventrally relative to its respective caudal ends, in lateral view. Such displacement reduces toward the caudal half of the series, with both cranial and caudal ends of the same centra in the similar level. Parapophyses were observed only in the two cranialmost trunk centra, similar to other theropods, which have parapophyses sitting ventral or on the same level of the neurocentral sutures around the trunk centra 1st to 4th^9,39^.

All sacral centra are platycoelous. The centra are short with articular facets as wide laterally as their craniocaudal length. Mid-centra are transversely constricted, bearing articular ends 1.7–2.0 times lateromedially wider than the mid-centra. The sacral centra are dorsoventrally low relative to the pre-sacral centra. Their articular facets are about 2.5 times broader lateromedially than tall, featuring a crescent-shape in cranial/caudal view. Its ventral surfaces are flattened in lateral view, as in *Masiakasaurus*. The caudalmost sacral has near circular outlines in cranial and caudal views. Its neural arch presents parapophyses closely attached to the transversal processes. The transversal process is dorsoventrally flat near the contact with the ilium but expands at the base to form the rib plate. The dorsocaudal surface of the neural arch bears large paired foramina on its left and right sides (Fig. 5f). A smaller subsidiary foramen lies lateral to the right foramen, in the caudalmost sacral neural arch. The neural spine is dorsoventrally reduced relative to *Elaphrosaurus* and Abelisauridae^3,39,41^. Its caudal surface bear a scar for interspinuous ligament on the dorsal half, while a postspinal groove lies on the ventral half.

The proximal caudal centra show a single shallow pleurocoel on their lateral surfaces. A single proximal caudal centrum shows two pleurocoels on its left lateral surface (its right lateral is hidden by matrix). The articular surfaces of the three putatively proximalmost centra are nearly circular with slightly concave dorsal and ventral edges in cranio-caudal views but become lateromedially flattened (height/width = 1.2) in the subsequent ones. Centra are craniocaudally longer than tall (2.0–2.6), as in noasaurines, but differ from the shortened centra of abelisaurids (about 0.7–2.0) and *Elaphrosaurus* (1.2–1.4). A single proximal caudal centrum shows a deep longitudinal sulcus on its ventral surface. Yet, some centra lack both groove and keel. Two weak laminae connect the centrum to diapophyses, differing from the three laminae observed in *Masiakasaurus*^9^.

The mid-distal tail centra are slender and show a shallow longitudinal groove. The transversal processes are rectangular. A small, short neural spine is borne at the caudal third of the craniocaudal axis of the arch. Acuminate and straight prezygapophyses project cranially. The distal vertebrae present simplified and rod-like neural arches. Deep longitudinal sulci are located on its ventral surfaces, whereas shallow longitudinal grooves reside on lateral surfaces. The zygapophyses exceed the centrum length proximally and distally especially in the most distal vertebrae.

The scapula is slender and long, being considerably longer than the humerus, a feature common to ceratosaurs. Both, rostrodorsal and caudoventral margins of the scapula are straight in lateral view, and parallel to each other. The distal end of the scapula is slightly expanded, featuring a rounded ‘boot’. The coracoid is rounded with an elongated process projected from its caudoventral margin. The caudal edge of the coracoid has a deep notch just above the glenoid. A low mound-like coracoid tubercle is present.

The left humerus, radius and ulna are proximodistally reduced, as expected for Ceratosauria. The humeral shaft and the proximal end are strongly flattened craniocaudally, differing from the globular pattern present in most ceratosaurians. The long axis of the proximal end of the humerus is displaced medially from that of the distal end by about 7°. The humeral head gently projects medially and bears a relatively small internal tuberosity. The greater tubercle does not reach the level of the proximal end, providing a lateral vacuity proximal to the greater trochanter, as in *Elaphrosaurus*^5^ and *Masiakasaurus*^6,8^. A faint deltopectoral crest protrudes cranially along the lateral border. In general, the humerus of *Berthasaura* is near similar to the referred humerus of *Vespersaurus* (MPCO.V 0006d)^11^, both in shape and size. However, the humerus of the *Berthasaura* differs from MPCO.V 0006d by: total length/midshaft width ratio higher than MPCO.V 0006d; proximal end more medially displaced relative to the distal end of the humerus; and lower (both craniocaudally and laterally) and less apically constrained deltopectoral crest.

The radial and ulnar lengths are less than a half of the humeral length. The radial and ulnar shafts are bowed. The distal end of the radius is 1.25 times wider than its proximal end in caudal view. The proximal articular surface of the ulna is faintly concave. The olecranon process is short and rounded. The proximal end of the ulna expands caudally toward the proximal end of the radius without significantly covering its proximal surface. The metacarpals are reduced in size and morphologically simplified in comparison to the metacarpals of *Masiakasaurus* and *Elaphrosaurus*, but similar to *Limusaurus*^4,7^ and *Eoabelisaurus*^41^. The metacarpal III is more gracile than the metacarpal II, but they do not differ substantially in length, similar to the manus of *Limusaurus*. The proximal articular surface is rounded and slightly convex. The shaft is straight, but a slight constriction is present in the ventral surface of the metacarpal. A single manual ungual was preserved (Fig. 5n). The shaft of this ungual is dorsoventrally tall and mediolaterally flattened, differing from the mediolaterally expanded manual claw of *Limusaurus*. A low median keel projects on the proximal articular surface, similar to *Noasaurus*^25^*.* A single dorsal vascular groove runs longitudinally on the proximal half of the claw. A low median ventral keel lies on the ventral surface on the mid-shaft to distal portion of the claw, resembling the condition of holotype of *Noasaurus* (PVL 4061)^25^.

The pelvic elements are unfused, as in *Limusaurus*^3,7^. The ilium is low (Fig. 5p), with a length four times its height, as coelophysoids, *Masiakasaurus*, and abelisaurids. The dorsal margin is straight and horizontal in lateral view, as in abelisaurids, *Limusaurus*, basal tetanureans and some paravians. The iliac blade is lateromedially flattened and strong vertically oriented, acquiring an almost laminar shape. The narrowed configuration resembles *Limusaurus*^7^, *Megapnosaurus*^17^, and ornithomimosaurian theropods^42^, but differs from most ceratosaurs and other theropods, whose iliac blades are dorsolaterally oriented or flare laterally. The supraacetabular crest has a triangular outline in dorsal view, as in *Vespersaurus*, but the apex of the supraacetabular crest faces ventrally, instead of lateroventrally. Caudally, the supraacetabular crest is continuous with the lateral brevis shelf, as typical for Ceratosauria, but unlike *Vespersaurus* the supraacetabular crest does not bifurcate into caudal and medial ridges^11^. In most theropods, the brevis fossa separates the lateral and medial brevis shelves, but both the medial brevis shelf and brevis fossa are reduced in *Berthasaura*. The brevis fossa is fully covered by lateral brevis shelf in lateral view.

The ischial articulation of the pubis is as mediolaterally wide as the iliac articulation, both corresponding to twice the surface of the acetabular margin in lateral view. This contrasts with a pubis referred to *Vespersaurus* (MPCO.V 0042)^11^, which shows proportionally wider ischial articulation. Both pubo-iliac and pubo-ischial articulations feature the peg-and-socket condition of ceratosaurs, with the ‘sockets’ located on the pubis. The iliac socket of the pubis lacks the medial and lateral walls, providing a concave outline in lateral view, as in MPCO.V 0042. An ovoid fossa to accommodate the prongs of ischium lies on the ischial articular facet.

In lateral view, the iliac articulation of the ischium is wider than the pubic articulation. The craniodorsal margin of the acetabulum is less dorsally inclined than *Vespersaurus*^11^, resembling the condition in *Masiakasaurus*^9^. In proximal view, iliac articulation exhibits the socket-like receptacle that articulates with the ischial peduncle of the ilium. The iliac articulation faces more cranioventrally relative to ischial acetabular margin than those of *Masiakasaurus* and *Vespersaurus*. This condition results from a hypertrophied dorsal end of the ischium over the ventral end in *Berthasaura leopoldinae*. This differs from the aforementioned noasaurids, in which both dorsal and ventral ends of the iliac articulation are equally expanded. The ventral margin of the ischial obturator plate projects caudally, forming a large, wing-like obturator process, as in *Eoabelisaurus*^41^. A deep incision separates the obturator process from the shaft. A ventromedially-oriented flange-like protuberance runs longitudinally through the ventral surface of the proximal shaft. This condition differs from *Vespersaurus*, which exhibits a flat ventral surface in the homologous region, and also from *Masiakasaurus*, which presents a smaller ridge on the same level of *Berthasaura*.

The femoral head is craniomedially directed, the typical ceratosaurian condition. The femoral shaft is straighter than those of most other noasaurids, but is similar in this respect to the Adamantina noasaurid femur^43^. A prominent cranial tuber protrudes from the cranioproximal margin of the femoral head. It proximally exceeds the proximal articular surface of the femur, while overhanging dorsally the cranial face of the femur. Distal to this tuber, a notch deeply separates the cranioproximal surface of the femoral head from the cranial trochanter (= anterior trochanter), as in coelophysoids^44^, but differing from other ceratosaurian femora from *Cemitério dos Pterossauros* Quarry^12^. This notch is not well developed in *Masiakasaurus* and *Elaphrosaurus*. A relatively low medial epicondylar crest protrudes at the craniomedial side of the femur through the distalmost fifth of the shaft, resembling the rounded crest of *Elaphrosaurus* both in shape and height.

The lateral margin of proximal articular surface of the tibia is displaced proximal to the medial margin. The cnemial crest is slightly elevated over the level of proximal articular surface, as in other ceratosaurs and non-coelurosaurian tetanurans. A thin fibular crest protrudes from the medial surface and extends distally, as in other ceratosaurs. The fibular crest is displaced from the articular surface of the tibia, similar to the condition of tetanurans^45^. It merges within the shaft few millimeters distal to the level of the cnemial crest. A remarkably deep and wide concavity is formed on the shaft, between the cnemial and fibular crests, occupying most of the craniomedial face. This concavity likely houses the proximal half of the fibula, similar to *Elaphrosaurus*. The tibial distal ends are not preserved in the specimen.

The proximal end of fibula is concave-convex in the medial–lateral aspect. The attachment scar for the *M. iliofibularis* consists of a low ridge on the lateral surface. In medial view, a shallow depression bears on the proximal end of the shaft. This depression is also present in *Deltadromeus*^46^, cf. “*Bahariasaurus*”^47^, and *Masiakasaurus*^9^, but it does not deeply incise the fibula as the latter taxon. A deep medial fossa is found immediately below this depression. It opens to the medial face, as in *Majungasaurus* and *Deltadromeus*, instead of caudomedially as in *Masiakasaurus*.

The phalanx is essentially similar to the isolated pedal phalanx 1 (MPCO.V0049) referred to *Vespersaurus*^11^.The pedal ungual (Fig. 5v) is lateromedially compressed. It is 2.25 times longer than its maximum height. It exhibits a single vascular groove on its lateral surface, instead the two grooves of abelisauroids. The vascular groove extends from three quarters of the dorsolateral surface and does not reach the level of the articular surface. It extends across a straight trajectory along the phalanx.

### Phylogenetic analysis

**Figure 6:**
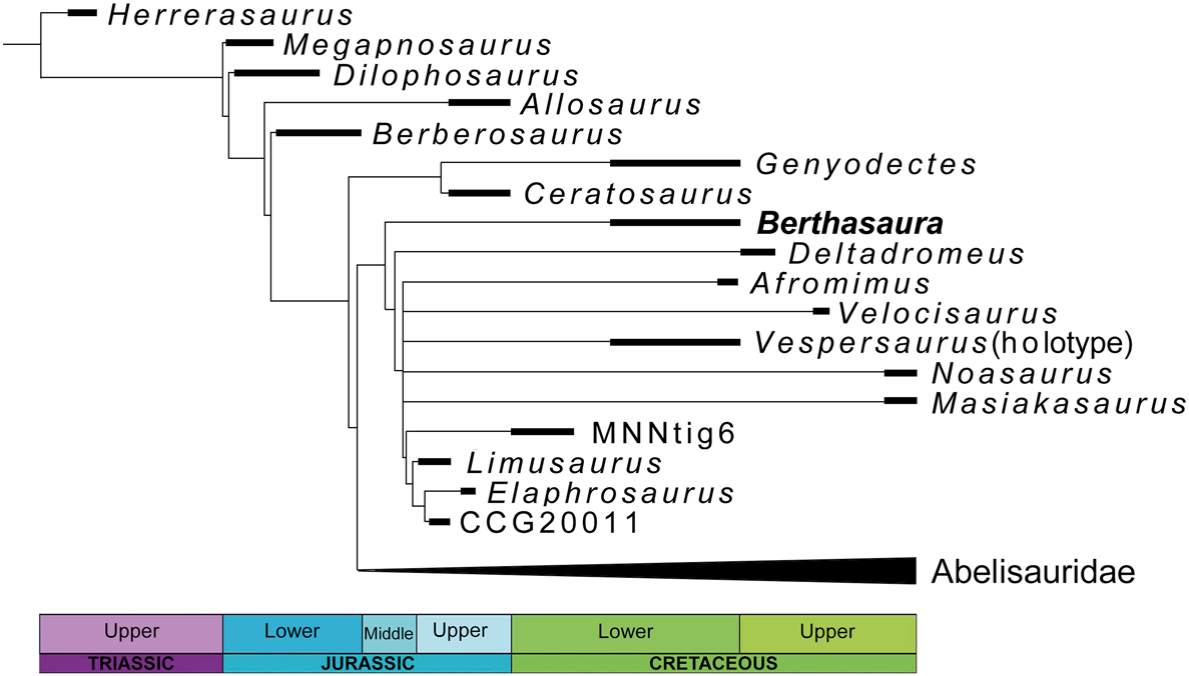
Phylogenetic relationship of *Berthasaura leopoldinae* gen. et sp. nov*.*, holotype (MN 7821-V). Time-calibrated reduced strict consensus trees of the 448 equally minimum-length trees with 454 steps each.

*Berthasaura* exhibits numerous features present in Ceratosauria^2,4^. The holotype MN 7821-V was scored in the character matrix of Rauhut and Carrano^5^, modified by Langer and colleagues^11^ (Fig. 6; Supplementary Data). The first analysis including all taxa resulted in more than 3,000 trees of minimum-length and 470 steps (CI = 0.504; RI = 0.725). The consensus tree is not well resolved (Fig. 6). The Bremer support was low for all nodes (= 1), except for Neotheropoda. *Berthasaura* nested as the basalmost noasaurid was recovered in most trees (see Supplementary Data for details). The conflict in the consensus tree is due to the unstability of abelisaurids *Dahalokely*, *Rahiolisaurus,* and *Kryptops*, and the noasaurids *Laevisuchus*, *Noasaurus*, *Vespersaurus*, and *Velocisaurus*. Removing *Dahalokely*, *Rahiolisaurus* and *Kryptops*, the phylogenetic analysis resulted 448 minimum-length trees of 454 steps (CI = 0.522; RI = 0.744). Regarding the strict consensus tree, *Berthasaura leopoldinae*, *Deltadromeus*, and *Laevisuchus* formed a polytomy at the base of Noasauridae, whereas *Masiakasaurus, Noasaurus, Vespersaurus*, *Afromimus* and Elaphrosaurinae formed a more inclusive polytomy (Fig. 6). *Berthasaura leopoldinae*, *Deltadromeus*, and *Laevisuchus* were placed outside “core noasaurids” (Elaphrosaurinae and “noasaurines”) in all trees. *Berthasaura leopoldinae* and *Deltadromeus* alternated as the basalmost Noasauridae. *Laevisuchus* and *Afromimus* were also recovered in different positions within Noasauridae. *Laevisuchus, Noasaurus*, and *Afromimus* were responsible for the instability within these nodes (Supplementary Data). Even excluding *Verspersaurus* from the analysis does not change the basal position of *Berthasaura*.

The following synapomorphies supported the inclusion of *Berthasaura leopoldinae* within Noasauridae: the postzygapophyses in mid-cervical vertebrae overhanging the centrum caudally (character 113:1); the low mid-cervical epipophyses with less than one-third of the height of the neural arch (character 116:0); the length of mid-cervical centra 2 to 3 times their height (character 117:1); the cranial articular surface of the trunk centra dorsoventrally compressed (character 123:1); length of the trunk centra more than 1.5 times their height (character 127:0); relatively wide spacing between the glenoid lip and caudoventral process on coracoid (character 152:1); and a medially opened fibular fossa on the medial aspect of the fibula. *Berthasaura* lacks the following characters that unite *Deltadromeus* and other noasaurids: low and rectangular neural spines of mid-caudal vertebrae (character 143:1), coracoid height more than 1.8 times its length (character 153:1), and stout humeral head (character 154:1). *Berthasaura* also lacks the double collateral grooves on the pedal ungual that unite “core noasaurids” (= Noasauridae excluding *Deltadromeus*). The reduced width of the shaft of metatarsal II relative to metatarsals III and IV represented an ambiguous synapomorphy that supported “core noasaurids”, but this anatomical region is not preserved in the *Berthasaura leopoldinae* holotype.

## Discussion

The *Cemitério dos Pterossauros* Quarry is a very interesting locality that became famous for being the first pterosaur bone-bed from Brazil, showing two quite distinct species^10,48^. Although the presence of dinosaurs was known right from the beginning of the studies concerning the specimens from this site^48^, the first dinosaur formally described was *Vespersaurus paranaensis*^11^ based on several isolated or partially associated elements. The holotype (MPCO.V 0065) consists of the centra of three dorsal, three sacral vertebrae, and three caudal vertebrae; a partial ilium and ischium; and a partially articulated pes that have been regarded as a potential chimera^10^. Even when all of the 47 further specimens referred to this species as paratypes are scored as a terminal entry, *Vespersaurus paranaensis* and *Berthasaura leopoldinae* were never recovered as close related taxa in the phylogenetic analyses.

Considering the holotype of *Vespersaurus paranaensis* (MPCO.V 0065), despite its fragmentary nature, there are several characters distinguishing these two noasaurids, most concerning the pelvis, which bears the sole common comparable elements. Among the most conspicuous features that differentiate these species are the mediolaterally flattened iliac blade, reduced medial brevis shelf, and the presence of a deep notch on caudal margin of ischial process producing a eminent and caudally-oriented prong which are present in *Berthasaura*. Comparisons of the the iliua indicate that the holotype of *Vespersaurus paranaensis* represents a slightly larger animal than the holotype of *Berthasaura leopoldinae*. If the assignment of one isolated tooth (MPCO.V 0020c) to *Vespersaurus* proves to be correct, then the edentulous condition of *Berthasaura* is another distinguishing feature between these noasaurids.

The most significant feature of *Berthasaura* is the edentulous condition which was only reported in more mature ontogenetic stages of *Limusaurus*^4^ among non-coelurosaurian theropods. In *Limusaurus* the teeth count reduces along the ontogeny of this species until the dorsal enclosure of the alveoli. *Limusaurus* completely lost its teeth around the third year of life, that corresponds to the ontogenetic stage 4^.^. Whereas ontogenetic truncation of odontogenesis was suggested to explain the series of transformations that generated the edentulism of *Limusaurus*, it is difficult to confirm whether the toothlessness in *Berthasaura* occurred via similar mechanism. Alternatively, the new Brazilian taxon might have never bore teeth, contrary to all other ceratosaurs, an hypothesis that is favored here. For the sake of discussion, if young individuals of *Berthasaura* ever had teeth, based on the available specimen they have lost them relatively earlier in ontogeny than *Limusaurus*. In any case, our phylogenetic analysis demonstrates that *Berthasaura* and *Limusaurus* are not closely related, suggesting that the loss of teeth evolved independently at least twice in noasaurid ceratosaurs.

**Figure 7:**
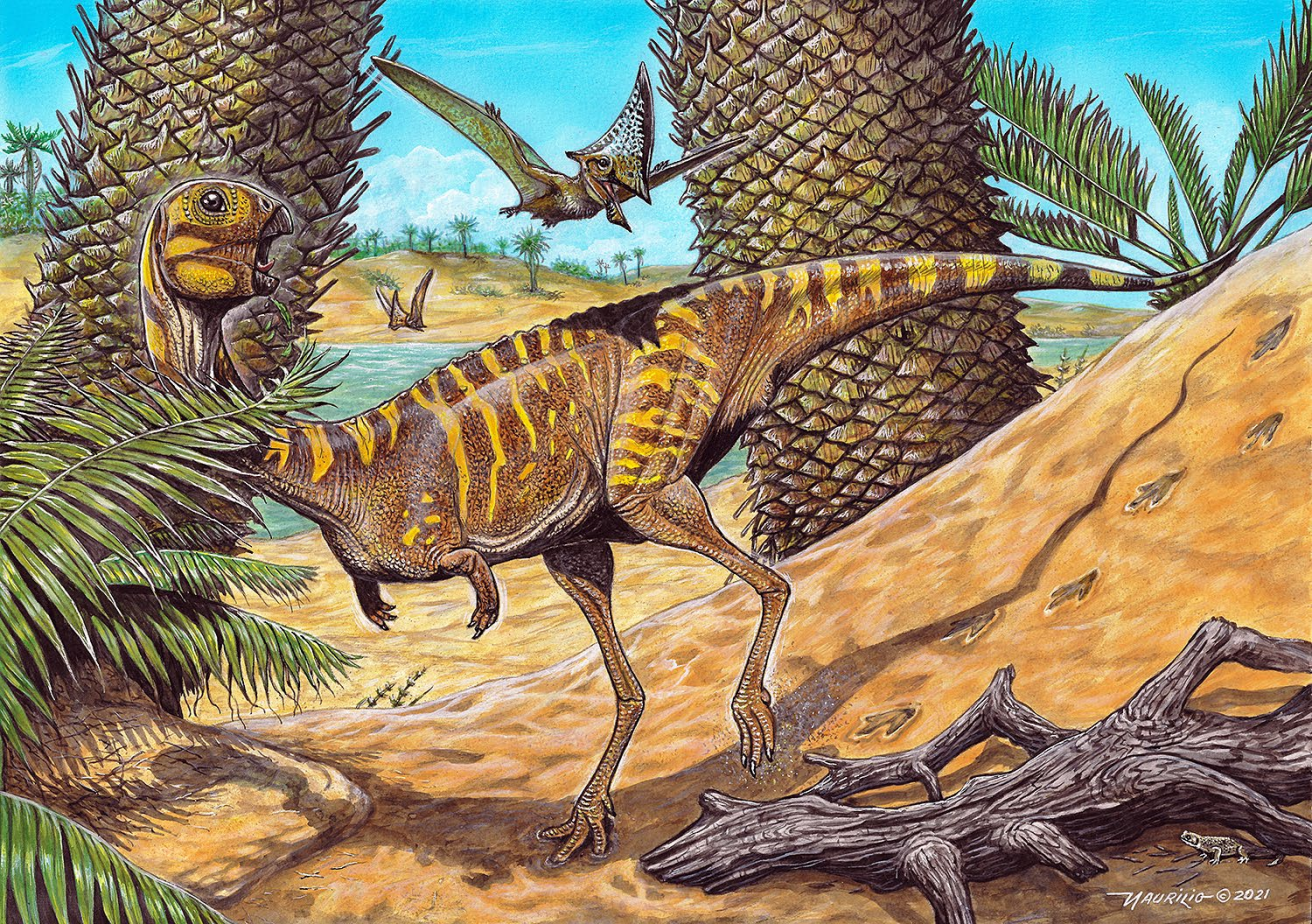
Life reconstruction of *Berthasaura leopoldinae* gen. et sp. nov*.* in the paleoenvironment represented by the “Cemitério dos Pterossauros” Quarry.

Gracile skull, edentulous rostrum, potential rhamphothecae, buccal cutting edge, ventral deflection of the dentary, and simplified biting surface are an amalgam of traits observed in *Berthasaura* that might be correlated to dietary specializations and are reported for other edentulous (or partially edentulous) dinosaurs, such as *Limusaurus*, ornithomimosaurians, oviraptorosaurians, therizinosaurians, early avians, ornithischians (*e.g., Hadrosaurus*), and even in poposauroidea psedosuchians (e.g., *Effigia okeeffeae*, *Shuvosaurus inexpectatus*) and chelonians, reinforcing repetitive evolution of such dietary traits among uncorrelated lineages^14,24,39–42,49–52^. However, it is problematic to infer the diet of fossil species based only on morphological data. Despite beaks being easily repurposed for faunivory along theropod evolution^50^, tooth loss and rhamphotheca alone are not reliable indicatives of herbivory^51^. Comparative anatomy of the skulls, the presence of gastroliths, and isotopic data have been used to infer herbivory in *Limusaurus*, *Incisivosaurus*, and some ornithomimosaurians with certain confidence^24,51^. No gastroliths, were recovered associated with *Berthasaura* and isotopic data are not available yet. In contrast, several herbivorous traits shared by *Berthasaura* and the aforementioned herbivorous taxa, suggest that *Berthasaura* might have been herbivorous (Fig. 7) or had, at least, omnivorous dietary preferences^49,50^.

In summary, *Berthasaura leopoldinae* is a nearly complete and well-preserved noasaurid that possesses unique anatomical features among ceratosaurs, particularly the edentulous rostrum. In addition, it comprises the second report of toothlessness among non-coelurosaurian theropods, alongside the Chinese noasaurid *Limusaurus inextricabilis*. A suite of jaw features diagnoses the new species, but the most notable of them are the premaxilla with a buccal cutting edge and an especially short dentary. A rhamphotheca might have been present (Fig. 7) and acted as a cutting edge potentially capable of slicing food. Nonetheless, the assignment of faunivory, herbivory, or omnivory for *Berthasaura leopoldinae* is limited based on available anatomical evidence, but numerous putative non-carnivorous-related traits are unequivocally present in the new species, increasing the potential of feeding modes among Ceratosauria. *Berthasaura leopoldinae* reveals that small-body and divergence in feeding habits may have occurred together across the earlier noasaurid evolutionary history. Finally, the *Cemitério dos Pterossauros* Quarry from the Goio Êre Formation in Southern Brazil is a site with the potential to provide valuable taxonomic and paleobiological information concerning Cretaceous vertebrate faunas of South America.

## Methods

### Material

The material here described as new species was collected under the regulation of the Departamento Nacional de Produção Mineral (DNPM) during fieldwork of 2011–2015 leading by a team of paleontologists from the Museu Nacional (UFRJ) and the CENPALEO (UnC). The specimen is housed in the Paleovertebrate Collection of the Museu Nacional under the collect number MN 7821-V.

### Nomenclatural acts

This published work and the nomenclatural acts it contains have been registered in ZooBank, the proposed online registration system for the International Code of Zoological Nomenclature. The ZooBank Life Science Identifiers (LSIDs) can be resolved, and the associated information viewed by appending the LSIDs to the prefix http://zoobank.org/. The LSID for this publication is urn:lsid:zoobank.org:pub:D34A4EC2-72BC-44ED-9DC5-0DC038B59D9B, for the genus *Berthasaura* urn:lsid:zoobank.org:act:BEEE1CD5-4F70-481C-AEE3-5C28A01FC5E5, and species *Berthasaura leopoldinae* urn:lsid:zoobank.org:act:20822433-A0CE-45DE-84A5-936295006BDE.

### Descriptions and comparisons

We used the directional terms ‘rostral’, ‘cranial’, and ‘caudal’ instead of ‘anterior’ and ‘posterior’. We follow the standardized morphological nomenclature of per example Wilson^53,54^ for saurischian vertebrae, Hendriczk^55^ for theropod maxillae, and Hendriczk, Araujo and Matheus^56^ for non-avian theropod quadrates.

In order to investigate the presence of alveoli or alveolar homologs, the dentary was subjected to computed microtomography scanning (µCT-Scan) imaging at Laboratório de Instrumentação Nuclear (LIN)—COPPE/UFRJ, using the high-power micro-focus tube of the GE v|tome|× 180/300 System. The experimental conditions were 100 kV and 80 µA. Data were exported in *.dcm format for the visualization in the free Software FIJI Image. The Bio-formats Exporter plugin was used for exporting data^57^.

### Phylogenetic analysis

To infer the phylogenetic relationships of *Berthasaura leopoldinae*, we used the character matrix of Rauhut and Carrano^5^, modified by Langer and colleagues^11^, excluding the paratypes of *Vespersaurus paranaensis* (see Supplementary Data), under equally weighted maximum parsimony in free software TNT 1.5^58^. We have also run the analysis scoring all specimens attributed to *Vespersaurus paranaensis* (holotype and paratypes^11^), which were regarded as a chimera^10^, as single terminal entry and the result has not affected the topology of the most parsimonious trees, always showing these two species distantly related (see Fig. 6 and Supplementary Data).

## Supplementary Information

Supplementary Information.

## References

[1] Tykoski RS, Rowe T, Weishampel DB, Dodson P, Osmólska H (2004). Ceratosauria. The Dinosauria.

[2] Carrano MT, Sampson SD (2008). The phylogeny of Ceratosauria (Dinosauria: Theropoda). J. Syst. Palaeontol..

[3] Filippi LS, Méndez AH, Valieri RDJ, Garrido AC (2016). A new brachyrostran with hypertrophied axial structures reveals an unexpected radiation of latest Cretaceous abelisaurids. Cretac. Res..

[4] Wang S (2017). Extreme ontogenetic changes in a ceratosaurian theropod. Curr. Biol..

[5] Rauhut OWM, Carrano MT (2016). The theropod dinosaur *Elaphrosaurus bambergi* Janensch, 1920, from the Late Jurassic of Tendaguru, Tanzania. Zool. J. Linn. Soc..

[6] Sampson SD, Carrano MT, Forster CA (2001). A bizarre predatory dinosaur from the Late Cretaceous of Madagascar. Nature.

[7] Xu X (2009). A Jurassic ceratosaur from China helps clarify avian digital homologies. Nature.

[8] Carrano MT, Sampson SD, Forster CA (2002). The osteology of *Masiakasaurus knopfleri*, a small abelisauroid (Dinosauria: Theropoda) from the Late Cretaceous of Madagascar. J. Vertebr. Paleontol..

[9] Carrano MT, Loewen MA, Sertich JJW (2011). New materials of *Masiakasaurus knopfleri* Sampson, Carrano, and Forster, 2001, and implications for the morphology of the Noasauridae (Theropoda: Ceratosauria). Smithson. Contrib. Paleobiol..

[10] Kellner, A. W. A., Weinschütz, L. C., Holgado, B., Bantim, R. A. M. & Sayão, J. M. A new toothless pterosaur (Pterodactyloidea) from Southern Brazil with insights into the paleoecology of a Cretaceous desert. *Anais da Academia Brasileira de Ciências* **91** (2019). 10.1590/0001-3765201920190768 31432888

[11] Langer MC (2019). A new desert-dwelling dinosaur (Theropoda, Noasaurinae) from the Cretaceous of south Brazil. Sci. Rep..

[12] de Souza GA (2020). Osteohistology and growth dynamics of the Brazilian noasaurid *Vespersaurus paranaensis* Langer et al., 2019 (Theropoda: Abelisauroidea). PeerJ.

[13] Wilson JA (2003). A new Abelisaurid (Dinosauria, Theropoda) from the Lameta Formation (Cretaceous, Maastrichtian) of India. Contrib. Mus. Paleontol..

[14] Kellner AWA (2019). Pterodactyloid pterosaur bones from cretaceous deposits of the Antarctic Peninsula. An. Acad. Bras. Cienc..

[15] Batezelli A, Ladeira FSB (2016). Stratigraphic framework and evolution of the Cretaceous continental sequences of the Bauru, Sanfranciscana, and Parecis basins, Brazil. J. S. Am. Earth Sci..

[16] Kellner AWA (2015). Comments on triassic pterosaurs with discussion about ontogeny and description of New Taxa. An. Acad. Bras. Cienc..

[17] Griffin CT (2018). Developmental patterns and variation among early theropods. J. Anat..

[18] Griffin CT (2021). Assessing ontogenetic maturity in extinct saurian reptiles. Biol. Rev..

[19] Brochu CA (1996). Closure of neurocentral sutures during crocodilian ontogeny: Implications for maturity assessment in fossil archosaurs. J. Vertebr. Paleontol..

[20] Ikejiri T (2012). Histology-based morphology of the neurocentral synchondrosis in *Alligator mississippiensis* (Archosauria, Crocodylia). Anat. Rec..

[21] Brissón Egli F, AgnolÍn FL, Novas F (2016). A new specimen of *Velocisaurus unicus* (Theropoda, Abelisauroidea) from the Paso Córdoba locality (Santonian), Río Negro, Argentina. J. Vertebr. Paleontol..

[22] Grillo ON, Delcourt R (2017). Allometry and body length of abelisauroid theropods: *Pycnonemosaurus nevesi* is the new king. Cretac. Res..

[23] Lautenschlager S, Witmer LM, Altangerel P, Zanno LE, Rayfield EJ (2014). Cranial anatomy of *Erlikosaurus andrewsi* (Dinosauria, Therizinosauria): New insights based on digital reconstruction. J. Vertebr. Paleontol..

[24] Barrett PM (2005). The diet of ostrich dinosaurs (Theropoda: Ornithomimosauria). Palaeontology.

[25] Bonaparte, J. F. & Powell, J. E. continental assemblage of tetrapods from the Upper Cretaceous beds of El Brete, northwestern Argentina (Sauropoda-Coelurosauria-Carnosauria-Aves). *Mémoires la Société géologique Fr.* 19–28 (1980).

[26] Ezcurra MD (2007). The cranial anatomy of the coelophysoid theropod *Zupaysaurus rougieri* from the Upper Triassic of Argentina. Hist. Biol..

[27] Madsen JH, Welles SP (2000). Ceratosaurus (dinosauria, theropoda) a Revised Osteology.

[28] Wang M, Hu H (2017). A comparative morphological study of the jugal and quadratojugal in early birds and their dinosaurian relatives. Anat. Rec..

[29] Carrano MT, Benson RBJ, Sampson SD (2012). The phylogeny of Tetanurae. J. Syst. Paleontol..

[30] Cerroni MA, Canale JI, Novas FE (2020). The skull of *Carnotaurus sastrei* Bonaparte 1985 revisited: insights from craniofacial bones, palate and lower jaw. Hist. Biol..

[31] Sampson SD, Witmer LM (2010). Craniofacial anatomy of *Majungasaurus crenatissimus* (Theropoda: Abelisauridae) from the Late Cretaceous of Madagascar. J. Vertebr. Paleontol..

[32] Canale JI, Scanferla CA, Agnolin FL, Novas FE (2009). New carnivorous dinosaur from the Late Cretaceous of NW Patagonia and the evolution of abelisaurid theropods. Naturwissenschaften.

[33] Currie PJ, Zhao X (1993). A new carnosaur (Dinosauria, Theropoda) from the Jurassic of Xinjiang, People’s Republic of China. Can. J. Earth Sci..

[34] Evers SW, Foth C, Rauhut OWM (2020). Notes on the cheek region of the Late Jurassic theropod dinosaur *Allosaurus*. PeerJ.

[35] Coria R, Salgado L (2000). A basal Abelisauria Novas, 1992 (Theropoda-Ceratosauria) from the Cretaceous of Patagonia, Argentina. Gaia.

[36] Elzanowski A, Peters DS, Mayr G (2018). Cranial morphology of the early Cretaceous bird Confuciusornis. J. Vertebr. Paleontol..

[37] Zheng X (2020). New information on the keratinous beak of *Confuciusornis* (Aves: Pygostylia) from two new specimens. Front. Earth Sci..

[38] Wang S (2017). Erratum: Heterochronic truncation of odontogenesis in theropod dinosaurs provides insight into the macroevolution of avian beaks. Proc. Natl. Acad. Sci. U. S. A..

[39] O’Connor PM (2010). The postcranial axial skeleton of *Majungasaurus crenatissimus* (Theropoda: Abelisauridae) from the Late Cretaceous of Madagascar. J. Vertebr..

[40] Brum AS, Machado EB, de AlmeidaCampos D, Kellner AWA (2018). Description of uncommon pneumatic structures of a noasaurid (Theropoda, Dinosauria) cervical vertebra from the Bauru Group (Upper Cretaceous). Brazil. Cretac. Res..

[41] Pol D, Rauhut OWM (2012). A middle Jurassic abelisaurid from Patagonia and the early diversification of theropod dinosaurs. Proc. R. Soc. B Biol. Sci..

[42] Osmólska H, Roniewicz E, Barsbold R (1972). A new dinosaur, *Gallimimus bullatus* n. gen., n. sp. (Ornithomimidae) from the Upper Cretaceous of Mongolia. Palaeontol. Pol..

[43] Martinelli AG (2019). Noasaurid theropod (Abelisauria) femur from the upper Cretaceous Bauru Group in Triângulo Mineiro (Southeastern Brazil). Cretac. Res..

[44] Nesbitt SJ (2011). The early evolution of archosaurs: Relationships and the origin of major clades. Bull. Am. Mus. Nat. Hist..

[45] Rauhut OWM, Cladera G, Vickers-Rich P, Rich TH (2003). Dinosaur remains from the lower Cretaceous of the Chubut Group, Argentina. Cretac. Res..

[46] Sereno PC (1996). Predatory dinosaurs from the Sahara and Late Cretaceous faunal differentiation. Science.

[47] Stromer E (1934). Ergebnisse der Forschungsreisen Prof. E. Stromers in den Wüsten Ägyptens. XIII. Dinosauria. Abhandlungen der Bayer. Akad. der Wissenschaften Neue Folge.

[48] Manzig PC (2014). Discovery of a rare pterosaur bone bed in a cretaceous desert with insights on ontogeny and behavior of flying reptiles. PLoS ONE.

[49] Button DJ, Zanno LE (2020). Repeated evolution of divergent modes of herbivory in non-avian Dinosaurs. Curr. Biol..

[50] Zanno LE, Makovicky PJ (2011). Herbivorous ecomorphology and specialization patterns in theropod dinosaur evolution. Proc. Natl. Acad. Sci. U. S. A..

[51] Lautenschlager S, Brassey CA, Button DJ, Barrett PM (2016). Decoupled form and function in disparate herbivorous dinosaur clades. Sci. Rep..

[52] Nesbitt S (2007). The anatomy of Effigia okeeffeae (Archosauria, Suchia), theropod-like convergence, and the distribution of related taxa. Bull. Am. Mus. Nat. Hist..

[53] Wilson JA (1999). A nomenclature for vertebral laminae in sauropods and other saurischian dinosaurs. J. Vertebr. Paleontol..

[54] Wilson JA, D’Emic MD, Ikejiri T, Moacdieh EM, Whitlock JA (2011). A nomenclature for vertebral fossae in sauropods and other saurischian dinosaurs. PLoS ONE.

[55] Hendrickx C, Mateus O (2014). *Torvosaurus gurneyi* n. sp., the largest terrestrial predator from Europe, and a proposed terminology of the maxilla anatomy in nonavian theropods. PLoS ONE.

[56] Hendrickx C, Aráujo R, Mateus O (2015). The non-avian theropod quadrate I: Standardized terminology with an overview of the anatomy and function. PeerJ.

[57] Linkert M (2010). Metadata matters: Access to image data in the real world. J. Cell Biol..

[58] Goloboff PA, Farris JS, Nixon KC (2008). TNT, a free program for phylogenetic analysis. Cladistics.

