# Supplementary material for: The first edentulous ceratosaur from South America
Source: Sci Rep. 2021 Nov 18;11:22281. doi: 10.1038/s41598-021-01312-4 (PMC8602317; doi:10.1038/s41598-021-01312-4)
Supplement: Supplementary file 1 — Supplementary Information. [file 41598_2021_1312_MOESM1_ESM.doc]

**The first edentulous ceratosaur from South America**

**Geovane Alves de Souza**^1,2^**, Marina Bento Soares**^2^**, Luiz Carlos Weinschütz**^3^, **Everton Wilner**^3^, **Ricardo Tadeu Lopes**^4^**, Olga Maria Oliveira de Araújo**^4^**, Alexander Wilhelm Armin Kellner**^2*^

^1^Programa de Pós-Graduação em Zoologia (PPGZoo), Museu Nacional/Universidade Federal do Rio de Janeiro. Quinta da Boa Vista s/n, São Cristóvão, CEP 20940–040, Rio de Janeiro, RJ, Brazil.

^2^Laboratório de Sistemática e Tafonomia de Vertebrados Fósseis (LAPUG), Departamento de Geologia e Paleontologia, Museu Nacional/Universidade Federal do Rio de Janeiro. Quinta da Boa Vista s/n, São Cristóvão, CEP 20940–040, Rio de Janeiro, RJ, Brazil.

^3^Centro Paleontológico da Universidade do Contestado (CENPALEO), Universidade do Contestado, Av. Presidente Nereu Ramos, 1071, Jardim Moinho, CEP 89.306-076, Mafra, SC, Brazil.

^4^Laboratório de Instrumentação Nuclear (LIN), Programa de Engenharia Nuclear /COPPE, Universidade Federal do Rio de Janeiro, Av. Horácio Macedo, Cidade Universitária, CEP 21941–450, Rio de Janeiro, RJ, Brazil.

*Corresponding Author:

Alexander Wilhelm Armin Kellner

Email address: kellner@mn.ufrj.br

**Supplementary Table S1 - Measurements (mm) of *Berthasaura leopoldinae*.**

| **Element** | **L** | **CC** | **ML** | **H** | **other measurements** |
| --- | --- | --- | --- | --- | --- |
| Right premaxilla | x | 14.1 | 0.9 | 21.1 | Height above the naris = 9.2 |
| Left premaxilla | x | 15.4 | 0.9 | 18.3* | Height above the naris = 9.1 |
| Left maxilla | x | 33.2 | 4* | ? | Height jugal ramus = 5 |
| Left nasal | x | 26.4* | 6.9 | 8.4 |  |
| Left frontal | x | 29.3 | 7.6 | ? |  |
| Left parietal | x | 19.7 | 0.7 | 11 |  |
| Right lacrimal | x | 20.1 | ? | 22.2 |  |
| Left lacrimal | x | 22.9 | 2.9 | 17.8 |  |
| Left postorbital (?) | x | 14.7 | ? | 17.8 |  |
| Left squamosal | x | 14* | ? | 11.2* |  |
| Left quadratic head | x | 6.2 | 5.7* | 14.9* |  |
| Right paraoccipital process | 19.2 | x | 3.1 | 4.6* |  |
| Left paraoccipital process | 19.2 | x | 3.1 | 6.5 |  |
| Endocranial case (prootic) | x | 10.2 | ? | 9.6 | Bone wall thickness = 1.7 |
| Basioccipital | x | 16 | ? | ? |  |
| Right palatine | x | 37.0 | 13.9 | ? |  |
| Left palatine | x | ? | 13.2 | ? |  |
| Left pterygoid (?) | x | 33.3 | ? | ? |  |
| Left ectopetygoid (?) | x | ? | 6.7 | ? |  |
| Right dentary |  | 33.5* | 2.9 | 11 | Simphyseal region width = 4.75 |
| Left dentary | x | 29.4* | 3.3 | 9.7 | Height at the level of EMF= 18.4 |
| Left splenial | x | 18.3 | ? | 8.1 |  |
| Left surangular | x | 32.2* | 2.5 | 15 | Height above the glenoid = 4 |
| Left retroarticular process | 9 | x | 4 | 2.2* |  |
| Left prearticular | x | 28.5* | ? | 5* |  |
| Left angular | x | 39.6 | ? | 2.9 |  |
| Hyoid | 59 | x | 2.5 | 2.6 |  |
| Intercentrum 1 | 3 | x | 6 | ? |  |
| Intercentrum 2 | 3 | x | 6 | ? |  |
| Axial centrum | 9 | x | 5 | 5 |  |
| Cranial cervical centrum (4th?) | 12 | x | 6 | 4 |  |
| Cranial cervical arch (4th?) | 18 | x | 11 | ? |  |
| Mid-cervical centrum (6th?) | ? | x | 7 | 5 |  |
| Mid-cervical arch (6th?) | ? | x | 15 | 9 |  |
| Mid-caudal cervical arch (7th?) | ? | x | 8 | 6 |  |
| Caudal cervical centrum (10th?) | 15 | x | 4 | 7 |  |
| Caudal cervical arch (10th?) | 20 | x | ? | 11 |  |
| Cervical rib (most complete shaft) | 48* | ? | ? | ? |  |
| Cervical rib (most complete shaft) | 53* | ? | ? | ? |  |
| 1st trunk neural arch | 16* | x | 31 | 11* |  |
| 1st trunk centrum | 13 | x | 8 | 6 |  |
| 2nd trunk neural arch | 20 | x | 26 | ? |  |
| 2nd trunk centrum | ? | ? | ? | ? |  |
| 3rd trunk centrum | 12 | x | 5 | 6 |  |
| 4th trunk neural arch | 15* | x | 38 | ? |  |
| 4th trunk centrum | ? | x | 7 | 7 |  |
| 5th trunk neural arch | 22.5* | x | 38 | ? |  |
| 5th trunk centrum | ? | x | 4 | 4 |  |
| Isolated trunk centrum (6th?) | 18 | x | 6 | 6.5 |  |
| Mid-trunk neural arch (7th?) | 16 | x | 30 | 7 |  |
| Mid-trunk centrum (7th?) | 19 | x | 4.5 | 8.9 | Mediolateral diameter at the proximal and distal ends = 8.5 |
| Mid-caudal trunk neural arch (8th?) | 20 | x | 14.8 | 12 |  |
| Mid-caudal trunk centrum (8th?) | 19.2 | x | 7.7 | 8 |  |
| Caudal trunk neural arch (9th?) | 20 | x | 34 | 12 |  |
| Caudal trunk centrum (9th?) | 19 | x | 8 | ? |  |
| Caudal trunk neural arch (10th?) | 23 | x | 11 | 6 |  |
| Caudal trunk centrum (10th?) | ? | ? | ? | ? |  |
| Caudal trunk neural arch (11th?) | 29 | x | 32 | 14 |  |
| Caudal trunk centrum (11th?) | ? | x | 12 | 9 |  |
| 1st left trunk rib | 72.3 | 2.9 | 4.5 | x |  |
| 2nd left trunk rib | 75.7 | ? | 4.27 | x | Capitulum length = 24.7 |
| 3rd left trunk rib | 78.1 | ? | 4.4 | x |  |
| 4th left trunk rib | 63.3 | ? | 3.4 | x |  |
| 5th left trunk rib | 80.7 | ? | 3.2 | x | Capitulum length = 13.8 |
| 9th left trunk rib fragment | ? | ? | 2.8 | x |  |
| 10th left trunk rib | ? | ? | 2.8 | x | Capitulum length = 15.2 |
| 1st sacral neural arch | 20 | x | 28 | 20 |  |
| 1sr sacral centrum | 16 | x | 11 | 8 | Mid-length width = 11 |
| 2nd sacral centrum | 15.4 | x | 15 | 9 | Mid-length width = 11 |
| 3rd sacral centrum | 16 | x | 17 | 18.5 | Mid-length width = 11 |
| 4th sacral centrum | 16 | x | 13 | 7 | Mid-length width = 11 |
| 5th sacral neural arch | 14 | x | 32 | 12 |  |
| 5th sacral centrum | 17 | x | 12 | 11 | Mid-length width = 11 |
| Isolated sacral neural arch (3rd or 4th?) | 25 | x | 26 | 13 |  |
| Isolated proximal tail centrum | 17 | x | 11 | 11 |  |
| 1st caudal neural arch | 14 | x | 46 | 15 |  |
| 1st caudal centrum | 18 | x | 12 | 6 |  |
| 2nd caudal neural arch | 22 | x | 46 | ? |  |
| 2nd caudal centrum | 16 | x | 10 | 10 |  |
| 3rd caudal neural arch | 19 | x | 30 | ? |  |
| 3rd caudal centrum | ? | x | 8 | 9 |  |
| 4 th caudal neural arch | 19 | x | 36 | ? |  |
| 4th caudal centrum | 15 | x | 5* | 9 |  |
| 5th caudal neural arch | 16 | x | 33 | ? |  |
| 5th caudal centrum | 16 | x | 6 | 6 |  |
| 6th caudal neural arch | ? | x | 34 | ? |  |
| 6th caudal centrum | 15 | x | 6 | 5 |  |
| Mid-caudal neural arch | 16 | x | 26 | ? |  |
| Mid-caudal centrum | 13 | x | 16 | 5 |  |
| Mid-caudal neural arch | 18 | x | 23 | 4* |  |
| Mid-caudal neural arch | 12 | x | 19 | 4 |  |
| Mid-caudal centrum | 12 | x | 6 | 6 |  |
| Distal caudal neural arch | 19 | x | 3* | 4 |  |
| Distal caudal centrum | 15 | x | ? | 5 |  |
| Distal caudal neural arch | 17* | x | 2* | 3 |  |
| Distal caudal centrum | 15 | x | 6 | 4 |  |
| Proximal chevron | 38.6 | 4.8 | 6.9 | x |  |
| Proximal chevron | 38 | ? | 6.5 | x |  |
| Proximal chevron | 27.1 | 4.9 | 4.8 | x |  |
| Mid-proximal chevron | 23 | 5.8 | ? | x |  |
| Mid-proximal chevron | 16.5 | 4.7 | ? | x |  |
| Left scapula | 84.6 | 11 | 2.5 | x | Glenoid heigth = 6 |
| Right scapula | 81 | 10 | 2.5 | x |  |
| Left coracoid | 28 | x | x | 24 | Length with caudoventral process = 35.6 |
| Humerus | 58 | 2 | 12 | x | Midshaft diameter = 6 |
| Radius | 31.8 | 2.9 | 1.8 | x | Craniocaudal diameter at the distal end = 6.8 |
| Ulna | 27.5 | 2.7 | 1.4 | x | Craniocaudal diameter at the proximal end = 6.45 |
| Manual phalanx II | 5.5* | x | 2.5* | ? |  |
| Manual phalanx III | 5.3 | x | ? | 1.8 |  |
| Manual ungual | 7.5 | x | ? | 2.3 |  |
| Ilium | 115 | x | 12 | 36 |  |
| Right femur | 132 | ? | 7* | x | Mediolateral diameter at the femoral head = 22.8 |
| Left femur | 116* | 11.7 | 10.6 | x | Mediolateral diameter at the femoral head = 20.7 |
| Right tibia fragment | 75.4* | ? | 11.3 | x |  |
| Right fibula | 102.3 | 4.2 | ? | x | Maximum craniocaudal diameter at the proximal end = 12.4 |
| Left fibula | ? | 6 | 16 | x |  |
| Right pubis | 113.9* | 5.9* | 3.9* | x |  |
| Left pubis | 108.3* | 6.9 | 3.9* | x | Mximum craniocaudal diameter at the obturator plate = 29.8 |
| Right ischium | 100.8* | 7.9 | ? | x | Maximum craniocaudal diameter at the obturator plate = 27.8 |
| Left ischium | 109.4* | 8.1 | ? | x | Maximum craniocaudal diameter at the obturator plate = 28.1 |
| Pedal digit II phalanx 1 | 24.3 | x | 3.8 | 5.2 | Mediolateral width at the proximal end = 7.9; and at the distal end = 6.3 |
| Pedal ungual | 15.7 | x | ? | 7.3 |  |

* Bone broken or partially hidden by the matrix

**Supplementary Data – Phylogenetic analyses.**

We scored the specimen MN 7821-V under the character matrix of Rauhut and Carrano^3^, modified by Langer and colleagues^4^, who added two extra characters to this dataset:

“*219 - Ischium, proximal end: pubic articular facet dorsoventrally deeper than that for the ilium (0); iliac articular facet dorsoventrally deeper than that for the pubis (1)”*, and;

“*220 - Metatarsal IV, shaft: uncompressed (0), lateromedially compressed (1) with respect to the shaft of metatarsal III”*;

We agree with the changes in the data matrix for *Limusaurus* and *Velocisaurus* proposed by the authors. However, since the *Cemitério dos Pterossauros* Quarry comprises a high sorted “*bonebed*” with disarticulated skeletons from different ontogenetic stages, which may lead to chimeric scoring, we ran alternative analyses, scoring *Vespersaurus* holotype as an Operational Taxonomic Unit (OTU) excluding the referred materials. As a test, we have also run the matrix with the original scoring of *Vespersaurus* by Langer et al.^4^ that did not change the position of both, *Vespersaurus* and *Berthasaura*. Matrixes were built in Mesquite 2.0^5^.

*Spinotropheus gauthieri* and its referred material MNN TIG6^6^ were scored as separate OTU, following Rauhut and Carrano^3^. Carrano and colleagues^7^ suggested that *Spinostropheus* holotype belongs to the tetanuran branch, whereas MNNTIG6 was recovered as Elaphrosaurinae or basal abelisauroid in different analyses^1,8^. The African theropod *Deltadromeus* is another putative noasaurid that lacks consensus regarding its phylogenetic positioning. Some authors recovered this species as an elaphrosaurine noasaurid, a basal ceratosaur^8^, basal coelurosaurian^9^ or neovenatorid allosauroid^10^. Carrano and colleagues^7,8^ stated that postcranial elements of the abelisaurid *Kryptops palaios* were found 15 meters from the holotipic maxilla and probably represent a carcharodontosaurid rather than Ceratosauria, which was corroborated by phylogenetic analyses performed by Wang and colleagues^1^. We kept all taxa *a priori* in our phylogenetic analyses to contrast our results to these assumptions in the light of new species herein scored.

Moreover, we added the recently described basal abelisaurid *Spectrovenator ragei*^11^ from Quiricó Formation of Brazil, and *Afromimus tenerensis*, from Elrhaz Fm., in Gadoufaoua locality, Niger^12^. *Spectrovenator* shows the combination of plesiomorphic and derivate features for Abelisauridae that may contribute to the character polarizing within Ceratosauria. *Afromimus* comprises several bones originally attributed to an ornithomimosaur, but subsequently found to be an abelisauroid, nested within an unresolved polytomy in Noasauridae^12^. In addition, *Caramillasaurus* was another recently described theropod, originally identified as a ceratosaur, but recent publications suggested that it actually represents a spinosaurid^13^. We added the scoring of *Spectrovenator* according Zaher and colleagues^11^ and scored *Afromimus* into the matrix. *Caramillasaurus* was not included in our analyses.

The basal saurischian *Herrerasaurus*, the coelophysoid *Megapnosaurus rhodesiensis*, the basal tetanurean *Dilophosaurus sinensis* (UCMP V 4214), the allosauroid *Allosaurus agilis* were considered as outgroup, following the Rauhut and Carrano^3^.

The resulting matrix comprises 38 taxa and 220 characters. The matrix was submitted to heuristic search of 10,000 replications of Wagner trees followed by Tree Bisection and Rearrangement (TBR) branch swapping algorithm, holding 5 trees for each replicate and radon seed equal ‘0’. Branches without length were collapsed after search using the line code ‘condense1;collapse;’ only for calculate Retention (RI) and Consistency (CI) indexes. Trees saved on RAM were subject to a second round of TBR branch swapping. We kept characters 33 and 165 as ordered (i.e., additive), as in Rauhut and Carrano^3^; and characters 33, 103, 104, 117, 138, 154, and 165, as in Langer and colleagues^4^. Bremer support was calculate using the Bremer script. Strict consensus was calculated using trees saved in RAM. Wildcard taxa were identified using InterPCR protocol, and their terminal entries were inactivated manually, and subject to new rounds of searches or only pruned *A posteriori* to improve the resolution in the consensus trees.

**Supplementary Data – Results of phylogenetic analyses.**

The first phylogenetic analysis resulted in more than 3,000 equally minimum-length trees. The first round of TBR analyzed 123,694,893 rearrangements and retained 1070 trees, with the best score hit 367 times out of 1000 replicates. The second TBR analyzed 61,626,996 rearrangements and retained 3736 trees. The best score did not vary between these two rounds, resulting in minimum-length trees with 470 steps (CI=0.504; RI= 0.725). Both CI and RI were lower than that obtained by Rauhut and Carrano^3^ (CI=0.55 and RI=0.73), probably due to the addition of fragmentary taxa, such as *Afromimus* and *Vespersaurus*. The Bremer support was low for all nodes (=1), except for the node Neotheropoda, which was more than one. Other authors found low support for many clades within Ceratosauria, especially Noasauridae^1,3,4,8,14^.

All minimum-length trees placed *Berthasaura leopoldinae* as a basalmost member of Noasauridae, being the sister-taxon of the clade comprised by the African *Deltadromeus* and the more derivate noasaurids. Both *Berthasaura*, *Deltadromeus*, and the highly fragmentary *Laevisuchus* represented the unique noasaurids placed outside the “Elaphrosaurinae-Nosaurinae” dichotomy (henceforward called “core noasaurids”, following Langer and colleagues^4^). Not rarely, the abelisauroid *Dahalokely* and the abelisaurid *Rahiolisaurus* yielded as sister-taxa of *Berthasaura leopoldinae* or in the basalmost position within Noasauridae. The position of *Dahalokely* as a sister-taxon of Noasauridae in some of these trees resembles the observations of Farke and Sertich^15^, however, it may result from conflict scoring, as other authors have advocated. *Spinostropheus* grouped with *Allosaurus*, reinforcing that this taxon represents a tetanuran theropod. *Eoabelisaurus* was yielded as the basalmost abelisaurid, followed by *Spectrovenator*, similarly to the topology recovered by Zaher and colleagues^11^. MNNTIG6 was recovered as the sister taxon of Elaphrosaurinae, contrasting with Rauhut and Carrano^3^ that recovered this specimen as the sister taxon of *Berberosaurus*, either at the base of Noasauridae or more closely related to the remaining noasaurids. *Afromimus* were recovered in several different positions within Noasaurinae. *Berthasaura*, *Laevisuchus*, and *Deltadromeus* were placed outside the core noasaurids in all trees. *Laevisuchus* was recovered in different positions: as the sister taxon of the core noasaurids, as basalmost noasaurid, or grouped with *Berthasaura* at the base of Noasauridae.

Regarding the consensus tree (Fig. 7), *Berberosaurus* was recovered as the basalmost ceratosaur. *Genyodectes* and *Ceratosaurus* were formed Ceratosauridae as sister taxon of Abelisauroidea. Surprisingly, all abelisaurid taxa were placed into a basal polytomy within Abelisauroidea, and only Noasauridae and a clade comprised by *Skorpiovenator*, *Ilokelesia*, and *Ekrixinatosaurus* were recovered as distinct clades. This topology differed from Rauhut and Carrano, which recovered only Elaphrosaurinae and Abelisauridae as distinct clades in the consensus tree. This indicates that the addition of the new taxon increased the resolution in the consensus of minimum-length trees. Among Noasauridae, only Elaphrosaurinae was recovered as a distinct clade. *Limusaurus* was recovered as the sister-taxa of *Elaphrosaurus* plus CCG 20011 within Elaphrosaurinae. The conflict in the consensus trees resulted from the unstable abelisaurids *Dahalokely*, *Rahiolisaurus* and *Kryptops*, and the noasaurids *Laevisuchus*, *Noasaurus*, *Vespersaurus*, and *Velocisaurus*.

Removing the problematic abelisaurids *Dahalokely*, *Kryptops*, and *Rahiolisaurus* a priori considerably reduced the number of minimum-length trees. The first round of TBR analyzed 42,274,926 rearrangements, totaling 448 Minimum-length trees of 454 steps. Forty-eight trees were retained for the second round of TBR, which analyzed 613,552 rearrangements, and retained the same number of trees. In comparison to the first analysis, the CI and RI increased to 0.522 and 0.744, respectively. *Berthasaura leopoldinae*, *Deltadromeus*, and *Laevisuchus* were placed outside the dichotomy Elaphrosaurinae-Noasaurinae in all trees. *Berthasaura leopoldinae* and *Deltadromeus* varied as the basalmost Noasauridae. *Laevisuchus* was recovered in different positions within Noasauridae: as the basalmost noasaurid, basal to *Deltadromeus* (but more derivate than *Berthasaura*), as a sister taxon of *Berthasaura*, or as the sister taxon of the ‘core noasaurids’ clade. *Afromimus* was recovered in different positions: as basalmost noasaurine, within the base of Noasaurinae only more derivate than *Masiakasaurus*, and as a sister species of either *Velocisaurus* or *Vespersaurus*.

Regarding the strict consensus tree, *Berthasaura*, *Deltadromeus*, and *Laevisuchus* comprised a polytomy in the base of Noasauridae. The noasaurids *Masiakasaurus, Noasaurus, Vespersaurus*, and *Afromimus* formed a polytomy along the Elaphrosaurinae. Again, *Laevisuchus, Noasaurus*, and *Afromimus* were responsible to provide the instability within these nodes.

*Laevisuchus* is known only from three cervical vertebrae and a trunk vertebra yielded from Late Cretaceous (Maastrichthian) deposits from Lameta Formation, in India. Due to its high incompleteness, *Laevisuchus* has been acting as wildcard taxa in all precedent analyses. However, several authors corroborated its status within Abelisauroidea, more precisely nested within Noasauridae, and outside core noasaurid dichotomy. There were not autapomorphies supporting *Laevisuchus* in all trees. The clade comprised of *Berthasaura* and *Laevisuchus* (in a minor number of trees), also lacked synapomorphy.

Pruning *Laevisuchus*, *Berthasaura leopoldinae* was found to be the basalmost noasaurid, only less derivate than *Deltadromeus* in the strict reduced consensus tree (SRCT). *Deltadromeus* was recovered as a sister group of remaining noasaurids. Noasaurinae remained as a polytomy formed by *Masiakasaurus*, *Vespersaurus*, *Velocisaurus*, and *Afromimus* in the SRCT. The relationships within Elaphrosaurinae followed the topologies of previous authors^1,3,8,14^. Pruning *Noasaurus* and *Afromimus* from the minimum-length trees, *Velocisaurus* and *Vespersaurus* recovered as sister taxa, and *Masiakasaurus* was yielded as sister species of this clade, similar to the topology of Langer and colleagues. The clade comprised by *Velocisaurus* and *Vespersaurus* were supported by a compressed shaft of metatarsal II relative metatarsals III and IV, meantime any autapomorphy was found to support either *Velocisaurus* or *Vespersaurus*.

When the type series of *Vespersaurus* was scored as a terminal entry (rather than only the holotype), the species remained supported by any autapomorphy, similarly to its original paper description. Despite the insertion of this entry terminal did not change the topology, it considerably increased the length and the number of the minimum-length trees (L=460), and reduced the resolution in the consensus tree. The Consistency and Retention indexes were slightly lower (CI=0.515 and RI=0.737) than that analysis comprising only the holotypic scoring. The clade comprised of *Vespersaurus* and *Velocisaurus* remained supported by the same synapomorphy. The decrease in the indexes and the support values may be explained by the character conflict, which probably resulted from the chimeric scoring of *Vespersaurus*. Most of the sediment blocks recovered from Cemitério dos Pterossauros Quarry exhibit an amalgam of pterosaur and theropod bones, ranging in different sizes and ontogenetic stages. The holotype and all the type series of *Vespersaurus* were found in an area of more than 500 square meters, with almost no evidence of association, and they were tentatively assigned to this species, according to the authors^4^. This is enough to biasing the delimitation of individuals in this site. We do not discard the plausibility of some elements attributed to *Vespersaurus* belong to *Berthasaura*, especially the humerus. A revaluation of the *Vespersaurus* hypodigm is beyond the scope of the present contribution. Meantime, we suggest a critical look at its type series until a further conclusion.

The following synapomorphies supported the inclusion of *Berthasaura leopoldinae* within Noasauridae: the postzygapophyses in mid-cervical vertebrae overhanging the centrum caudally; the low epipophyses with less than one-third of the height of the neural arch; the length of mid-cervical centra 2 to 3 times their height; the cranial articular surface of the trunk centra dorsoventrally compressed; length of the trunk centra more than 1.5 times their height; relatively wide spacing between the glenoid lip and caudoventral process on coracoid; and a medially opened fibular fossa on the medial aspect of the fibula. The long list of synapomorphies contrasts with the relatively lower nodal support values and indexes calculated, even when fragmentary taxa were ignored.

The following synapomorphies supported *Berthasaura leopoldinae*: single prong on rostral end of splenial; maximal length of metacarpus less than 15% of the length of humerus; and rounded medial epicondyle of femur. Some trees also recovered a postzygodiapophyseal lamina in mid-cervical vertebrae reduced, indicated by a low ridge in parts.

The position of *Berthasaura* as the sister taxon of *Deltadromeus* and the more derived noasaurids was supported by the absence of what follows: low and rectangular neural spines of mid-tail vertebrae, coracoid height more than 1.8 times its length, and stout humeral head. *Berthasaura* also lacks the double collateral grooves on pedal ungual that united the clade of core noasaurids. The reduced width of the shaft of metatarsal II relative to metatarsals III and IV represented an ambiguous synapomorphy that supported these derived noasaurids, but it was not preserved in the *Berthasaura* holotype.

Because the positioning of *Deltadromeus* within Noasauridae still is a matter of debate, it was excluded a priori for final analysis. The absence of *Deltadromeus* did not affect the main topology but increased the CI, and RI (L=434, CI=0.541 and RI=0.752). The Bremer support increased for two in Ceratosauria, Abelisauroidea, and Carnotaurini nodes, however, no improvement occurred for Noasauridae. Some trees recovered *Rahiolisaurus* as a basal abelisauroid or basal as a noasaurid, whereas a minor number of trees yielded *Velocisaurus* either as the sister-taxon of *Berthasaura leopoldinae*, or more basal than it. The holotypic *Vespersaurus* and *Velocisaurus* were responsible for the conflict in the consensus tree. Finally, the absence of *Deltadromeus* reduced the resolution of Noasauridae in the consensus tree.

**Supplementary Data – List of synapomorphies**

The following list of common synapomorphies is based on the optimization of all minimum-length trees after pruning the unstable taxa *Noasaurus*, *Afromimus*, *Laevisuchus*, *Dahalokely*, *Rahiolisaurus*, and *Kryptops*.


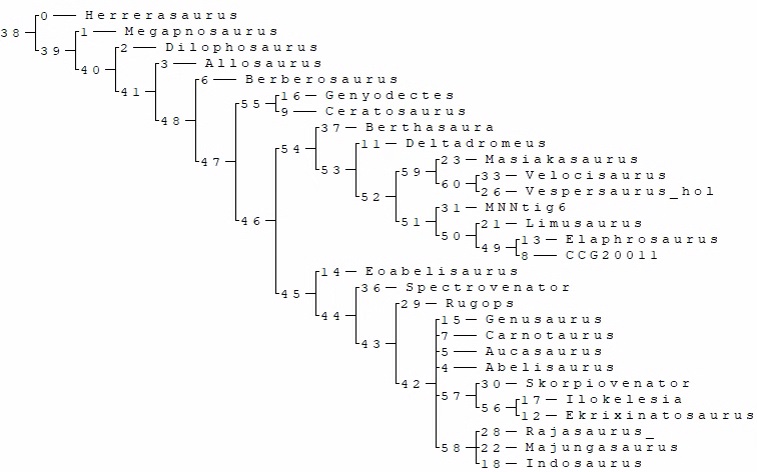


(Node numbers refer to nodes in consensus)

Herrerasaurus :

All trees:

No autapomorphies:

Megapnosaurus :

All trees:

Char. 10: 0 --> 1

Char. 108: 0 --> 1

Char. 116: 1 --> 2

Char. 118: 0 --> 1

Char. 123: 0 --> 1

Char. 133: 0 --> 1

Char. 151: 0 --> 1

Char. 169: 0 --> 1

Char. 173: 0 --> 1

Char. 175: 0 --> 1

Char. 176: 0 --> 1

Char. 177: 0 --> 1

Char. 179: 0 --> 1

Char. 180: 0 --> 1

Char. 196: 0 --> 1

Char. 197: 0 --> 1

Char. 204: 0 --> 1

Char. 208: 0 --> 1

Char. 212: 0 --> 1

Dilophosaurus :

All trees:

Char. 23: 0 --> 1

Char. 86: 0 --> 1

Char. 89: 0 --> 1

Char. 135: 0 --> 1

Allosaurus :

All trees:

Char. 24: 0 --> 1

Char. 37: 0 --> 1

Char. 103: 1 --> 0

Char. 104: 01 --> 2

Char. 107: 0 --> 1

Char. 139: 1 --> 0

Char. 149: 0 --> 1

Char. 168: 1 --> 0

Char. 181: 0 --> 1

Char. 202: 1 --> 2

Abelisaurus :

Some trees:

Char. 31: 1 --> 0

Char. 40: 0 --> 1

Char. 52: 1 --> 0

Char. 60: 1 --> 0

Aucasaurus :

All trees:

Char. 30: 1 --> 0

Some trees:

Char. 45: 1 --> 0

Berberosaurus :

All trees:

Char. 119: 0 --> 1

Carnotaurus :

All trees:

Char. 110: 0 --> 1

Char. 122: 0 --> 1

Char. 194: 0 --> 1

CCG20011 :

All trees:

Char. 123: 1 --> 0

Char. 125: 1 --> 0

Ceratosaurus :

All trees:

Char. 4: 0 --> 1

Char. 82: 0 --> 1

Deltadromeus :

All trees:

Char. 160: 1 --> 0

Char. 197: 1 --> 0

Ekrixinatosaurus :

All trees:

Char. 32: 1 --> 0

Char. 109: 1 --> 0

Elaphrosaurus :

All trees:

No autapomorphies:

Eoabelisaurus :

All trees:

Char. 21: 0 --> 1

Char. 36: 0 --> 1

Char. 39: 0 --> 1

Char. 154: 1 --> 0

Char. 213: 1 --> 0

Char. 218: 1 --> 0

Genusaurus :

All trees:

Char. 95: 2 --> 1

Char. 196: 1 --> 2

Char. 197: 1 --> 0

Genyodectes :

All trees:

No autapomorphies:

Ilokelesia :

All trees:

Char. 107: 1 --> 0

Indosaurus :

All trees:

No autapomorphies:

Limusaurus :

All trees:

Char. 153: 1 --> 0

Char. 168: 1 --> 0

Char. 173: 1 --> 0

Char. 178: 1 --> 0

Char. 179: 1 --> 0

Char. 181: 0 --> 1

Char. 184: 1 --> 0

Char. 185: 1 --> 0

Char. 190: 1 --> 0

Majungasaurus :

All trees:

Char. 136: 0 --> 1

Masiakasaurus :

All trees:

No autapomorphies:

Vespersaurus_hol :

All trees:

No autapomorphies:

Rajasaurus_ :

All trees:

Char. 122: 0 --> 1

Char. 131: 1 --> 0

Char. 177: 1 --> 0

Char. 213: 1 --> 0

Rugops :

All trees:

No autapomorphies:

Skorpiovenator :

All trees:

Char. 179: 1 --> 0

MNNtig6 :

All trees:

Char. 107: 1 --> 0

Char. 113: 1 --> 0

Char. 115: 0 --> 1

Char. 117: 1 --> 0

Char. 119: 0 --> 1

Char. 122: 1 --> 0

Char. 126: 0 --> 1

Velocisaurus :

All trees:

No autapomorphies:

Spectrovenator :

All trees:

No autapomorphies:

Berthasaura :

All trees:

Char. 79: 1 --> 0

Char. 164: 1 --> 2

Char. 196: 1 --> 0

Node 39 :

All trees:

No synapomorphies

Node 40 :

All trees:

Char. 32: 1 --> 2

Char. 77: 0 --> 1

Char. 102: 1 --> 2

Char. 159: 0 --> 1

Char. 202: 0 --> 1

Char. 210: 0 --> 1

Node 41 :

All trees:

Char. 60: 0 --> 1

Char. 61: 0 --> 1

Char. 62: 0 --> 1

Char. 78: 0 --> 1

Char. 98: 0 --> 1

Char. 99: 0 --> 1

Char. 100: 0 --> 1

Char. 112: 1 --> 0

Char. 113: 0 --> 1

Char. 114: 1 --> 2

Char. 115: 0 --> 1

Char. 116: 1 --> 0

Char. 152: 1 --> 0

Char. 195: 0 --> 1

Char. 200: 0 --> 1

Char. 205: 0 --> 1

Char. 206: 0 --> 1

Char. 207: 0 --> 1

Char. 209: 0 --> 1

Char. 217: 0 --> 1

Node 42 :

All trees:

Char. 20: 0 --> 1

Char. 21: 0 --> 1

Char. 22: 0 --> 1

Char. 28: 0 --> 1

Char. 47: 0 --> 1

Node 43 :

All trees:

Char. 13: 0 --> 1

Char. 26: 0 --> 1

Char. 88: 0 --> 1

Node 44 :

All trees:

Char. 1: 0 --> 1

Char. 19: 0 --> 1

Char. 24: 0 --> 1

Char. 30: 0 --> 1

Char. 31: 0 --> 1

Char. 33: 0 --> 1

Char. 45: 0 --> 1

Char. 54: 0 --> 1

Char. 87: 0 --> 1

Some trees:

Char. 141: 0 --> 1

Char. 198: 0 --> 1

Node 45 :

All trees:

Char. 44: 0 --> 1

Char. 135: 0 --> 1

Char. 137: 1 --> 2

Char. 140: 0 --> 1

Char. 167: 0 --> 1

Char. 214: 0 --> 1

Node 46 :

All trees:

Char. 3: 0 --> 1

Char. 9: 0 --> 1

Char. 11: 0 --> 1

Char. 67: 0 --> 1

Char. 75: 0 --> 1

Char. 106: 0 --> 1

Char. 107: 0 --> 1

Char. 109: 0 --> 1

Char. 127: 0 --> 1

Char. 137: 0 --> 1

Char. 138: 0 --> 1

Char. 145: 0 --> 1

Char. 147: 0 --> 1

Char. 150: 0 --> 1

Char. 156: 0 --> 1

Char. 160: 0 --> 1

Char. 162: 0 --> 1

Char. 164: 0 --> 1

Char. 184: 0 --> 1

Node 47 :

All trees:

Char. 95: 1 --> 2

Char. 165: 0 --> 1

Node 48 :

All trees:

Char. 103: 1 --> 2

Char. 130: 0 --> 1

Char. 196: 0 --> 1

Char. 203: 0 --> 1

Node 49 :

All trees:

Char. 103: 2 --> 1

Char. 120: 0 --> 1

Node 50 :

All trees:

Char. 106: 1 --> 0

Char. 114: 1 --> 0

Char. 144: 0 --> 1

Node 51 :

All trees:

Char. 95: 2 --> 1

Char. 104: 1 --> 0

Char. 127: 1 --> 0

Char. 145: 1 --> 0

Node 52 :

All trees:

Char. 211: 0 --> 1

Char. 214: 0 --> 1

Node 53 :

All trees:

Char. 142: 2 --> 1

Char. 152: 0 --> 1

Char. 153: 0 --> 1

Node 54 :

All trees:

Char. 112: 0 --> 1

Char. 115: 1 --> 0

Char. 116: 0 --> 1

Char. 122: 0 --> 1

Char. 126: 1 --> 0

Char. 151: 0 --> 1

Char. 202: 1 --> 2

Node 55 :

All trees:

Char. 91: 0 --> 1

Char. 92: 0 --> 1

Char. 93: 0 --> 1

Node 56 :

All trees:

Char. 104: 1 --> 2

Node 57 :

All trees:

Char. 37: 0 --> 1

Char. 41: 0 --> 1

Char. 118: 0 --> 1

Node 58 :

All trees:

Char. 66: 0 --> 1

Node 59 :

All trees:

Char. 182: 0 --> 1

Char. 212: 0 --> 1

Node 60 :

All trees:

Char. 219: 0 --> 1

**Supplementary Data – Map of synapomorphies.**

Character 80: Prongs at anterior end of splenial: one (0); two (1).


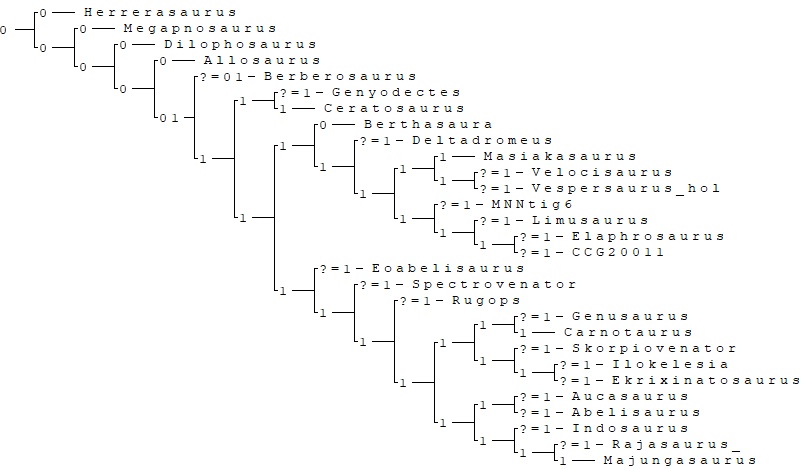


Character 165: Ratio between maximal length of metacarpus and length of humerus: more than 23% (0); 15–23% (1); less than 15% (2).

**
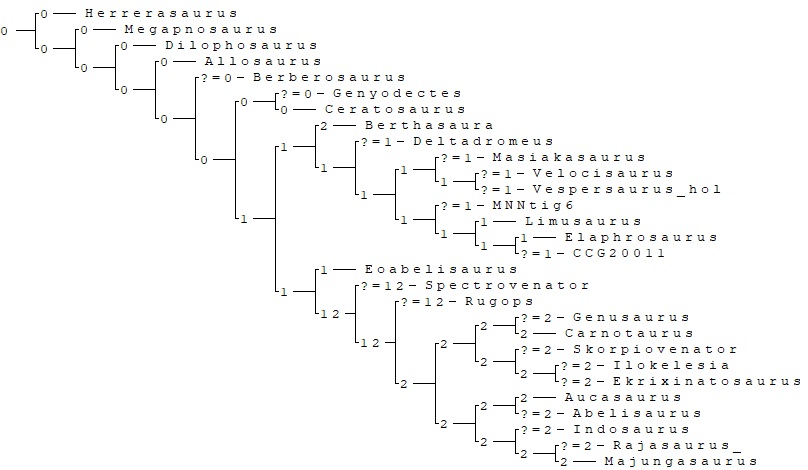
**

Character 197: Development of medial epicondyle of femur: rounded (0); ridge (1); long flange (2).

**
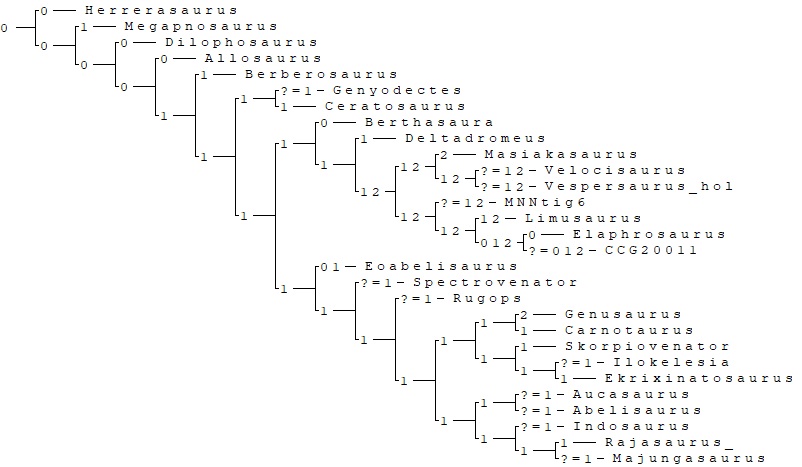
**

**Supplementary Data – Character list.**

1. External surface of maxilla and nasal: smooth (0); sculptured (1)^8^.

2. External surface of postorbital, lacrimal, and jugal: smooth (0); sculptured (1)^8^.

3. Maxillary process of premaxilla: well developed (0); reduced to a short triangle or absent (1) (Rauhut and Carrano^3^ modified from Carrano and Sampson^8^).

4. Subnarial foramen: enclosed (0); reduced/open dorsally (1)^8^.

5. Height/length ratio of premaxilla ventral to external naris: 0.5–2.0 (0); >2.0 (1)^8^.

6. Proportions/presence of the anterior ramus of the maxilla: absent (0); anteroposteriorly long (1); or tall and blunt (2)^8^.

7. Ascending process of the maxilla: posterodorsally directed, with strongly convex anterior and concave posterior margin (0); or subvertical, with almost straight anterior and posterior margins (1) (Rauhut and Carrano^3^ modified from Bonaparte and Novas^16^).

8. Extension of ascending process of maxilla: large, reaching posteriorly to more than half the length of the maxilla (0); reduced, posterodorsal tip ends well before the half-length of the maxilla (1) (Rauhut and Carrano^3^ modified from Canale and colleagues^17^).

9. Facet for nasal articulation on maxilla: shallow, anterolateral (0); socket, lateral (1)^8^.

10. Palatal process of maxilla: long and ridged (0); reduced, small, and blunt (1) (Rauhut and Carrano^3^ modified from Carrano and Sampson^8^).

11. Anteroventral border of antorbital fenestra: graded or stepped (0); demarcated by raised ridge (1)^8^.

12. Antorbital fossa: extends to the anterior margin of the ascending process, at least in its dorsal part (0); separated from the anterior margin of the ascending process (1) (Rauhut and Carrano^3^ modified from Canale an colleagues^17^).

13. Ventral portion of antorbital fossa: present on maxilla (0); strongly reduced or absent (1) (Rauhut and Carrano^3^ modified from Carrano and Sampson^8^).

14. Anteroposterior length of maxillary–jugal contact, relative to total maxilla length: less than 40% (0); more than 40% (1)^8^.

15. Inclination of maxilla–jugal contact: low, less than 45° from the horizontal (0); steep, more than 45° from the horizontal (1)^17^.

16. Nasal–nasal contact in adults: separate (0); partly or fully fused (1)^8^.

17. Row of foramina on dorsal nasal surface: absent (0); present (1)^8^.

18. Posterior narial margin: fossa (0); laterally splayed hood (1)^8^.

19. Location of nasal–frontal contact relative to highest point of orbit: anterior (0); directly above (1)^8^.

20. Condition of prefrontal in adults: separate (0); partly or completely fused (1)^8^.

21. Frontals unfused (0); or fused (1)^17^.

22. Frontal–parietal contact in adults: separate (0); fused at least on the dorsal surface (1) (Rauhut and Carrano^3^ modified from Carrano and Sampson^8^).

23. Skull roof dorsoventral thickness: thin, relatively flat (0); thickened (1) ^8^.

24. Skull roof ornamentation: none (0); midline (1); lateral (2)^8^.

25. Arrangement of bones along dorsal margin of orbit: postorbital and lacrimal separated by frontal, which forms part of orbital rim (0); contact between postorbital and lacrimal that excludes frontal from orbital rim (1)^8^.

26. Small fenestra in the skull roof placed between prefrontal, frontal, postorbital, and lacrimal: absent (0); or present (1)^6,17^.

27. Knob-like dorsal projection of parietals and supraoccipital: absent (0); or present (1)^8^.

28. Development of median parietal skull table: flat, broad (0); narrow, with sagittal crest (1)^8^.

29. Size and elevation of nuchal wedge and parietal alae: moderate (0); tall and expanded (1)^8^.

30. Size of infratemporal fenestra: subequal to size of orbit or smaller (0); considerably enlarged, larger than orbit and especially expanded ventrally (1) (Rauhut and Carrano^3^ modified from Rauhut^18^).

31. Postorbital suborbital flange: absent (0); present (1)^8^.

32. Anteroposterior length of postorbital relative to height: markedly less (0); equal to or greater (1)^8^.

33. Length of the posterior process of the postorbital: less than half of the length of the anterior process (0); more than half, but less than the length of the anterior process (1); longer than the anterior process (2) (Rauhut and Carrano^3^ modified from Canale and colleagues^17^) (ordered).

34. Orientation of posterior edge of postorbital: vertical (0); strongly sloped anteroventrally at an angle of 25° or more (1) (Rauhut and Carrano^3^ modified from Carrano and Sampson^8^).

35. Jugal process of the postorbital: with distinct kink in posterior margin at the beginning of the jugal facet (0); jugal facet continuous with posterior margin of the dorsal part of the postorbital (1)^19^.

36. Anterior margin of ventral process of the postorbital: straight or slightly concave (0); strongly concave (1) (Rauhut and Carrano^3^ modified from Canale and colleagues^17^).

37. Posterior margin of the ventral (jugal) process of the postorbital: convex in at least its dorsal part (0); straight or slightly concave over its entire length (1) (Rauhut and Carrano^3^ modified from Canale and colleagues^17^).

38. Dorsal margin of postorbital strongly inflated and bulked: absent (0); or present (1)^17^.

39. Lateral depression on the posterodorsal part of the junction of the postorbital processes: present (0); absent (1)^19^.

40. Morphology of anteroventral portion of ventral process of the postorbital: confluent with remainder of process (0); step and fossa present (1)^8^.

41. Morphology of dorsalmost postorbital–squamosal contact: smooth (0); knob (1)^8^.

42. Angle between dorsal margin of upper temporal arc (formed by the posterior process of postorbital and anterior process of squamosal) and dorsal skull roof: less than 45° (0); or more than 45° (1) (Rauhut and Carrano^3^ modified from Canale and colleagues^17^).

43. Appearance of postorbital–squamosal contact in lateral view: contact edges visible (0); edges covered by dermal expansions (1)^8^.

44. Anterior process of lacrimal: includes antorbital fossa and rim (0); antorbital fossa only (1)^8^.

45. Lacrimal fossa: exposed laterally (0); covered by dermal ossifications (1)^8^.

46. Suborbital process of lacrimal: absent (0); present (1)^8^.

47. Morphology of lacrimal along dorsal orbit rim: flat (0); raised brow or shelf (1)^8^.

48. Constriction of ventral ramus of lacrimal: present (0); absent (1) (Rauhut and Carrano^3^ modified from Canale and colleagues^17^).

49. Posterior expansion of ventral part of lacrimal: present, ventral part of posterior margin of lacrimal concave (0); strongly reduced or absent, ventral part of posterior margin straight or slightly convex (1) (Rauhut and Carrano^3^ modified from Canale and colleagues^17^).

50. Morphology of jugal–maxilla contact: slot or groove (0); lateral shelf (1)^8^.

51. Morphology of jugal–lacrimal articulation: simple joint (0); overlapping and pocketed (1)^8^.

52. Minimal height of jugal below the orbit: onethird or less of the height of the orbit (0); half the height of the orbit or more (1) (Rauhut and Carrano^3^ modified from Canale and colleagues^17^).

53. Ventral margin of the posterior half of the jugal: straight or only slightly convex (0); strongly convex (1) (Rauhut and Carrano^3^ modified from Canale and colleagues^17^).

54. Relative lengths of posterior jugal prongs: upper prong much shorter than lower prong (0); both prongs subequal in length (1)^8^.

55. Squamosal contribution to nuchal crest: absent or minimal (0); present and broad (1)^8^.

56. Quadrate flange of squamosal: wraps around quadrate head (0); ends posterior to quadrate head (1)^8^.

57. Dorsoventral proportions of quadratojugal prongs for jugal: narrow (0); deep (1)^8^.

58. Overlap of quadratojugal onto quadrate posteriorly: absent (0); present (1)^8^.

59. Quadrate foramen: present (0); absent (1)^8^.

60. Ossification of interorbital region: weak or absent (0); extensive (1)^8^.

61. Morphology of trigeminal foramen: single (0); partly or fully split (1)^8^.

62. Vagal canal opening: through otoccipital (0); onto occiput (1)^8^).

63. Depth of basisphenoid recess: shallow (0); deep (1)^8^.

64. Shape of opening for basisphenoid recess: ovoid (0); teardrop-shaped (1)^8^.

65. Depth of indentation between basal tubera and basisphenoid processes: deep notch (0); shallow embayment (1)^8^.

66. Medial fossa ventral to occipital condyle: absent (0); present (1)^8^.

67. Size of dorsal groove on occipital condyle: wide (0); narrow (1)^8^.

68. Orientation of basioccipital–basisphenoid suture: oblique (0); horizontal (1)^8^.

69. Depth of median ridge on supraoccipital: less than width (0); greater than width (1)^8^.

70. Morphology of jugal process of palatine: tapered process, triradiate palate (0); expanded process, tetraradiate palatine (1)^8^.

71. Pocket on ectopterygoid flange of the pterygoid: absent (0); present (1^8^.

72. Shape of pterygoid articulation with basipterygoid process: tab-like (0); acuminate (1)^8^.

73. Arrangement of jugal and pterygoid processes of ectopterygoid: oblique (0); parallel (1)^8^.

74. Proportions of ectopterygoid: gracile (0); robust (1)^8^.

75. Ventral excavation into ectopterygoid: absent (0); fossa (1); groove (2)^8^.

76. Size of external mandibular fenestra: small to moderate (0); large (1)^8^.

77. Position of anterior end of external mandibular fenestra relative to last dentary tooth: posterior (0); ventral (1)^8^.

78. Horizontal ridge on lateral surface of surangular below mandibular joint: weak or moderate (0); strong (1)^8^.

79. Contour of posterior edge of splenial: straight (0); curved or notched (1)^8^.

80. Prongs at anterior end of splenial: one (0); two (1)^8^.

81. Morphology of dentary–surangular articulation just above external mandibular fenestra: small notch (0); large socket (1)^8^.

82. Shape of articulated dentary rami in dorsal view: V–shaped (0); U–shaped (1)^8^.

83. Ventral margin of dentary: straight or only slightly convex (0); strongly convex anteroposteriorly (1)^17^.

84. Position of lateral dentary groove: at or above mid-depth (0); in ventral half (1)^8^.

85. Position of posterior end of posteroventral process of dentary relative to posterior end of posterodorsal process: far posterior (0); directly ventral (1)^8^.

86. Arrangement of premaxillary tooth carinae: nearly symmetrical, on opposite sides (0); more asymmetrical, both on lingual side (1)^8^.

87. Number of maxillary teeth: more than 12 (0); 12 or fewer (1)^8^.

88. Tooth curvature: substantially curved mesial and distal profiles, with apex positioned distal to distal profile (0); or apex centrally positioned, with mesial profile exhibiting strong curvature and distal profile straight or very slightly curved (1) (Rauhut and Carrano^3^ modified from Smith and colleagues^17,20^).

89. Surface texture of paradental plates: smooth (0); vertically striated or ridged (1)^8^.

90. Visibility of paradental plates in medial view: widely exposed (0); obscured (1)^8^.

91. Medial groove in paradental plates exposing replacement teeth: present (0); absent (1)^8^.

92. Pronounced size difference between premaxillary and maxillary teeth: absent (0) present (1) (Rauhut, 2004b).

93. Transverse flattening of lateral maxillary teeth: moderate (0); extreme (1)^21,22^.

94. Size of largest maxillary tooth: less (0) or more (1) than height of the dentary^21,22^.

95. Neural arch pneumaticity: moderate (0); extreme (1)^8^.

96. Internal structure of presacral vertebrae: solid (0); camerate (1); camellate (2)^8^.

97. Atlantal epipophysis: short and triangular in lateral outline (0); strongly elongate and rod-like (1) (Rauhut and Carrano^3^ modified from Canale and colleagues^17^).

98. Length of axial epipophyses: moderate (0); long (1)^8^.

99. Morphology of axial spinopostzygapophyseal lamina: weakly concave (0); deeply invaginated (1)^8^.

100. Development of axial diapophyses: weak, nubbin (0); prominent, pendant (1)^8^.

101. Axial pleurocoels: absent (0); present (1)^8^.

102. Postzygodiapophyseal lamina in axis: absent (0); present (1)^17^.

103. Anterior pleurocoel in postaxial cervical vertebrae: absent (0); fossa (1); foramen (2) (Rauhut and Carrano^3^ modified from Gauthier, 1986) (ordered).

104. Posterior pleurocoel in postaxial presacral vertebrae: absent (0); fossa only (1); fossa with pneumatic foramen (2)^8^. (ordered).

105. Anterior articular surface of postaxial cervical vertebrae: concave (0); flat (1); convex (2) (Rauhut and Carrano^3^ modified from Rauhut^18^).

106. Shape of postaxial cervical zygapophyses: anteroposteriorly elongated or ovate (0); mediolaterally elongated with lateral half greatly expanded anteroposteriorly (1)^15^.

107. Demarcation of dorsal surface of neural arch from diapophyseal surface in anterior cervical vertebrae: gently sloping (0); ridge (1)^8^.

108. Ratio between anterior width and height of middle and posterior cervical centra: less than 1.3 (0); more than 1.3 (1) (Rauhut and Carrano^3^ modified from Rauhut^18^).

109. Anteroposterior position of cervical neural spines: posterior half of centrum (0); anterior half of centrum (1)^8^.

110. Ventral keel on anterior cervicals: present (0); faint or absent (1)^8^.

111. Anterior prongs on postaxial cervical epipophyses: absent (0); present (1)^8^.

112. Development of pre- and postspinal fossae in postaxial cervical vertebrae: narrow (0); broad (1)^8^.

113. Postzygapophysis in mid-cervical vertebrae: placed at least partially over the posterior end of the neural arch pedicle and does not overhang the centrum posteriorly (0); posterior to the neural arch pedicle and overhangs the centrum posteriorly (1)^3,17,23^.

114. Position of cervical zygapophyses: close to midline (0); placed far laterally (1)^8^.

115. Morphology of anterior cervical epipophyses: low, blunt (0); long, thin (1); long, robust (2)^8^.

116. Height of mid-cervical epipophyses: low, less than one-third of the height of the neural arch (as measured from the dorsal margin of the centrum to the lateral edge of the postzygapophysis) (0); high, more than half the height of the neural arch (1)^3^.

117. Length/height ratio of mid-cervical centra: less than 2 (0); 2–3 (1); more than 3 (2) (Rauhut and Carrano^3^ modified from Carrano and Sampson^8^) (ordered).

118. Height of postaxial cervical neural spines: moderate or tall (0); short (1)^8^.

119. Postzygodiapophyseal lamina in mid-cervical vertebrae: present, and connects the diapophysis with the postzygapophysis (0); reduced, indicated by a low ridge in parts (1) (Rauhut and Carrano^3^ modified from Canale and colleagues^17^: character 57).

120. Accessory fossa on dorsal surface of postaxial cervical transverse processes: present (0); absent (1)^8^.

121. Constriction of posterior cervical vertebrae: moderate, centrum width at mid-length half the width at posterior end or more (0); extreme, mid-centrum width one-third or less (1)^3^.

122. Anterior pneumatic recess in anterior dorsals: absent (0); present (1) (modified from Rauhut^18^).

123. Shape of anterior articular surface of centrum in vertebrae D1 and D2: subcircular (0); dorsoventrally compressed, width/height ratio 1.15 or more (1) (Rauhut and Carrano^3^ modified from Farke and Sertich^15^).

124. Shape of dorsal transverse processes in dorsal view: rectangular (0); triangular (1)^8^.

125. Height of dorsal parapophyses: slightly elevated from centrum (0); project far laterally (1)^8^.

126. Paradiapophyseal lamina: absent, weak (0); pronounced (1)^8^.

127. Dorsal vertebral centrum length relative to height: more than 1.5 (0); less than 1.5 (1) (modified from Rauhut^18^).

128. Infradiapophyseal fossa of middle and posterior dorsal vertebrae: undivided (0); divided by paradiapophyseal lamina (1)^15^.

129. Orientation of prezygapophyses in posterior half of dorsal vertebral series in anterior view: dorsal or dorsomedial (0); dorsolateral (1)^15^.

130. Number of sacral vertebrae: five or less (0); six or more (1) (Rauhut and Carrano^3^ modified from Carrano and Sampson^8^).

131. Transverse dimensions of mid-sacral centra relative to other sacrals: equivalent (0); constricted (1)^8^.

132. Orientation of ventral margin of mid-sacral centra: horizontal (0); arched (1)^8^.

133. Dorsal edge of sacral neural spines: as thin as remainder of spine (0); thickened (1)^8^.

134. Condition of sacral neural spines in adults: separate (0); fused (1)^8^.

135. Pneumaticity of sacral neural spines: weak or absent (0); well developed (1)^8^.

136. Neural spines of anterior caudal vertebrae in comparison to last dorsal vertebrae: approximately of equal anteroposterior length (0); two thirds or less the length of last dorsal neural spines (1) (Rauhut and Carrano^3^ modified from Carrano and Sampson^8^).

137. Longitudinal ventral groove in anterior caudal vertebrae: present (0); absent (1) (Rauhut and Carrano^3^ modified from Rauhut^18^).

138. Centrodiapophyseal laminae in anterior midcaudal vertebrae: absent (0); only anterior centrodiapophyseal lamina present (1); anterior and posterior centrodiapophyseal laminae present as low, rounded ridges (2) (Rauhut and Carrano^3^ modified from Rauhut^18^) (ordered).

139. Proportions of anterior caudal neural arch base relative to mid-centrum proportions: smaller (0); equal or greater (1) (Rauhut and Carrano^3^ modified from Carrano and Sampson^8^).

140. Hyposphene–hypantrum articulations in anterior to mid-caudal vertebrae: absent (0); present (1)^17^.

141. Distal morphology of anterior to mid-caudal transverse processes: tapering (0); expanded posteriorly (1) (Rauhut and Carrano^3^ modified from Carrano and Sampson^8^).

142. Anterior process at distal end of anterior to midcaudal transverse processes: absent (0); present (1) (Rauhut and Carrano^3^ modified from Carrano and Sampson^8^).

143. Neural spines of mid-caudals: inclined posteriorly (0); low and rectangular (1); anteroposteriorly short and vertical (2) (Rauhut and Carrano^3^ modified from Rauhut^18^).

144. Proximodistal length of anterior caudal transverse processes: less than 1.4 times the length of caudal centra (0); more than 1.4 times the length of caudal centra (1) (Rauhut^18^).

145. Contact between cervical vertebrae and cervical ribs in adults: separate (0); fused (1)^8^.

146. Wing-like process at the base of the anterior cervical rib shafts: absent (0); present (1)^8^.

147. Bifurcate cervical rib shafts: absent (0); present (1)^8^.

148. Large vertical depression on lateral side of scapula above the glenoid: absent (0); present (1)^19^.

149. Distal expansion of scapula: present (0); absent (1) (Gauthier, 1986).

150. Relative width of scapular blade: broad, more than twice glenoid depth (0); narrow, less than twice glenoid depth (1)^8^.

151. Development of posteroventral process on coracoid: moderate (0); pronounced (1)^8^.

152. Spacing between glenoid lip and posteroventral process of coracoid: close, less than half of the length of the glenoid (0); wide, more than half of the length of the glenoid (1) (Rauhut and Carrano^3^ modified from Carrano and Sampson^8^).

153. Ratio of coracoid height (as measured over the glenoid) and coracoid length: less than 1.8 (0); more than 1.8 (1) (Rauhut and Carrano^3^ modified from Carrano and Sampson^8^).

154. Shape of humeral head in proximal view: transversely elongate, more than two times wider than long anteroposteriorly (0); stout, less than two times wider than long (1); globular (2) (Rauhut and Carrano^3^ modified from Carrano and Sampson^8^) (ordered).

155. Shape of distal humeral condyles: rounded (0); flattened (1)^8^.

156. Placement of humeral greater tubercle: proximally, usually confluent with proximal articular surface (0); offset distally, separated from articular surface further than the medial tuberosity (1) (Rauhut and Carrano^3^ modified from Carrano and Sampson^8^).

157. Posterolateral tubercle on proximal part of the humerus: absent or poorly developed as a low mound (0); well-developed, sharply defined tubercle (1)^24^.

158. Humerus: S–shaped in lateral view (0); straight (1)^18^.

159. Humerus in anterior view: with a concave or straight lateral margin and a mildly concave medial margin (0); with a convex lateral and strongly concave medial margin (1) (Rauhut and Carrano^3^).

160. Longitudinal torsion of humeral shaft: absent (0); present (1)^8^.

161. Size of deltopectoral crest: prominent flange (0); reduced (1)^8^.

162. Length of humerus relative to femur length: more than one-third (0); less than one-third (1)^8^.

163. Length of radius: more than half the length of the humerus (0); equal to or less than half the length of the humerus (1)^18^.

164. Distal articular surface of radius: flat or slightly concave (0); strongly convex (1) (Rauhut and Carrano^3^ modified from Canale and colleagues^17^: character 90).

165. Ratio between maximal length of metacarpus and length of humerus: more than 23% (0); 15–23% (1); less than 15% (2) (ordered) (Rauhut and Carrano^3^ modified from Pol and Rauhut^19^) (ordered).

166. Distal end of metacarpal I: strongly asymmetrical, lateral condyle considerably higher dorsoventrally and more distally expanded than medial condlye (0); asymmetry reduced, lateral and medial condyles of subequal size or indistinct (1)^3^.

167. Ventromedial expansion of medial distal condyle of metacarpal II: present (0); absent (1)^19^.

168. Pronounced rim on proximal border of extensor fossa on metacarpal II and metacarpal III: absent (0); present (1)^3^.

169. Distal articular end of metacarpal III in dorsal view: broad, with two condyles that are more or less symmetrical (0); broad, lateral condyle extends further distally than medial condyle (1); reduced in width, single convex articular surface (2)^3^.

170. Metacarpal IV: slender, width of mid-shaft less than half of the width of mid-shaft of metacarpal II (0); robust, width more than half of that of metacarpal II (1)^19^.

171. Relative length of manual phalanx II–1: more than twice width (0); less than twice width (1) (Rauhut and Carrano^3^ modified from Carrano and Sampson^8^).

172. Constricted neck between articular ends of manual phalanges in digits II and III: present (0); absent, phalanges very stout and broad (1) (Rauhut and Carrano^3^ modified from Pol and Rauhut^19^).

173. Gynglimoidal distal articular surface of proximal manual phalanges: well developed, condyles separated by deep notch (0); reduced, notch weak or absent (1) (Rauhut and Carrano^3^ modified from Canale and colleagues^17^: character 93).

174. Contacts between pelvic elements in adults: separate (0); at least partially fused (1)^8^.

175. Ratio of length of femur to length of ilium: more than 1.3 (0); less than 1.1 (1) (Rauhut and Carrano^3^ modified from Canale and colleagues^17^: character 82).

176. Posterior width of iliac brevis fossa: subequal to anterior width (0); twice anterior width (1)^8^.

177. Lateral brevis shelf in ilium: directed mainly ventrally, postacetabular blade of the ilium faces mainly laterally (0); flares laterally, postacetabular blade of the ilium faces dorsolaterally (1)^3^.

178. Morphology of lateral ilium between supraacetabular crest and brevis shelf: gap (0); continuous (1)^8^.

179. Anterior margin of ilium: faces anteriorly or anterodorsally (0); faces anteroventrally (1)^19^.

180. Shape of posterior margin of iliac postacetabular process: convex (0); undulating (1)^8^.

181. Shape of dorsal margin of central iliac blade: convex (0); straight (1) (Rauhut and Carrano^3^ modified from Carrano and Sampson^8^).

182. Relative sizes of iliac–pubic and iliac–ischial articulations: subequal (0); iliac–pubic articulation anteroposteriorly larger (1)^8^.

183. Relative proximodistal length of pubic and ischial peduncle: pubic peduncle considerably longer than ischial peduncle (0); peduncle of subequal length, or ischial peduncle longer (1)^19^.

184. Orientation of ischial peduncle of ilium: posteroventrally inclined (0); vertical (1)^19^.

185. Shape of anterior margin of iliac preacetabular process: rounded (0); undulating (1)^8^.

186. Anteroventral lobe of iliac preacetabular process: absent (0); present (1)^8^.

187. Contact between pubic apices: separate (0); contacting (1)^8^.

188. Morphology of contact between pubis and ilium: planar (0); peg-and-socket (1)^8^.

189. Morphology of dorsal surface of pubic boot on midline: convex (0); concave (1)^8^.

190. Notch ventral to obturator process on ischium: absent (0); present (1)^8^.

191. Morphology of distal ischium: rounded, separate (0); expanded, with triangular boot (1) (Rauhut and Carrano^3^ modified from Carrano and Sampson^8^).

192. Morphology of contact between ischium and ilium: planar (0); peg-and-socket (1)^8^.

193. Proportions of limb bones: moderate to gracile (0); robust (1)^8^.

194. Dimorphism in hindlimb morphology: absent (0); present (1)^8^.

195. Fourth trochanter of femur: developed as a high flange or crest (0); reduced to a low ridge (1)^17,25^.

196. Morphology of anterolateral muscle attachments on proximal femur: continuous trochanteric shelf (0); distinct lesser trochanter and attachment bulge (1)^8^.

197. Development of medial epicondyle of femur: rounded (0); ridge (1); long flange (2)^8^.

198. Morphology and orientation of femoral tibiofibularis crest: narrow, longitudinal (0); broad, oblique (1)^8^.

199. Cnemial crest: distal end subequal or smaller in proximodistal width to shaft of crest (0); distal end expanded proximodistally (1) (Rauhut and Carrano^3^ modified from Carrano and Sampson^8^).

200. Expansion of cnemial crest (as measured from the level of the tibial shaft): ratio tibial length/cnemial crest >6.5 (0); ratio tibial length/cnemial crest <6.5 (1) (Rauhut and Carrano^3^ modified from Canale and colleagues^17^: character 99).

201. Shape of distal tibia in distal view: rounded (0); mediolaterally elongate (1)^8^.

202. Anterior side of distal end of tibia: with marked step, demarcating the border of the ascending process of the astragalus (0); flat (1)^18^.

203. Development of fibular fossa on medial aspect of proximal fibula: posterior groove (0); posteriorly open fossa (1); medially open fossa (2)^8^.

204. Size of iliofibularis tubercle on fibula: moderate (0); large (1)^8^.

205. Contact between fibula and ascending process of astragalus in adults: separate (0); fused (1)^8^.

206. Morphology of astragalar ascending process: blocky (0); laminar (1)^8^.

207. Orientation of astragalar distal condyles: ventral (0); anteroventral (1) (Rauhut and Carrano^3^ modified from Carrano and Sampson^8^).

208. Horizontal groove across the anterior face of the astragalar condyles: absent or weak (0); pronounced (1)^8^.

209. Contact between astragalus and calcaneum in adults: separate (0); fused (1)^8^.

210. Development of astragalar articular surface for distal end of fibula: large, dorsal (0); reduced, lateral (1)^8^.

211. Height of the ascending process of the astragalus relative to depth of astragalar body: less or equal (0); greater (1)^8^.

212. Width of shaft of metatarsal II relative to widths of metatarsals III and IV: subequal (0); reduced (1)^8^.

213. Proximal articular surface of metatarsal II: subequal in width to that of metatarsals III and IV (0); two-thirds or less the width of metatarsals III or IV (1) (Rauhut and Carrano^3^ modified from Carrano and Sampson^8^: character 148).

214. Distal end of metatarsal IV: strongly asymmetrical, medial condyle much broader and more distally expanded than lateral condyle (0); more symmetrical, condyles subequal (1) (Rauhut and Carrano^3^ modified from Canale and colleagues^17^: character 112).

215. Morphology of lateral and medial grooves on pedal unguals: single (0); double (1)^8^.

216. Mediolateral symmetry of pedal digit II ungual: symmetrical (0); asymmetrical (1)^8^.

217. Ventral surface of pedal unguals with a well developed flexor tubercle, and usually flat (0); or without flexor tubercle and with a ventral depression in its proximal end (1)^17,26^.

218. Length of pedal digit phalanges I–1 + I–2 relative to III–1: greater (0); less than or equal (1)^8^.

219. Ischium, proximal end: pubic articular facet dorsoventrally deeper than that for the ilium (0); iliac articular facet dorsoventrally deeper than that for the pubis (1)^4^.

220. Metatarsal IV, shaft: uncompressed (0), lateromedially compressed (1) with respect to the shaft of metatarsal III^4^.

**Supplementary Data – Nexus file.**

BEGIN TAXA;

TITLE Taxa;

DIMENSIONS NTAX=38;

TAXLABELS

*Herrerasaurus Megapnosaurus Dilophosaurus Allosaurus Abelisaurus Aucasaurus Berberosaurus Carnotaurus* CCG20011 *Ceratosaurus Dahalokely Deltadromeus Ekrixinatosaurus Elaphrosaurus Eoabelisaurus Genusaurus Genyodectes Ilokelesia Indosaurus Kryptops Laevisuchus Limusaurus Majungasaurus Masiakasaurus Noasaurus* Composite_Vesp *Vespersaurus*_hol *Rahiolisaurus Rajasaurus Rugops Skorpiovenator* MNNtig6 *Spinostropheus*_type *Velocisaurus* USNM6415 *Afromimus Spectrovenator Berthasaura*

;

END;

BEGIN CHARACTERS;

TITLE Character_Matrix;

DIMENSIONS NCHAR=220;

FORMAT DATATYPE = STANDARD GAP = - MISSING = ? SYMBOLS = " 0 1 2 3 4 5 6 7 8 9 A B C D E F G H J K M N P Q R S T U V W X Y Z a b c d e f g h j k m n p q r s t u";

MATRIX

*Herrerasaurus* 000000000001100000000000000000001001000000000000000000?0000000000000000000100000000000000000100000000000000000000010000000001?00000000?000000000001100?000000000000100000000000000000000000000000000000-000000000000000000Me

*Megapnosaurus*  00000100001000000000000000000000100000000000000000000000000000000100000000100000000000000000000100000011000010001010211001010000000001001001000000000011000000000000000110001011101101000000100010011000000100010001?000010D

*Dilophosaurus*  00000000?000000??000??0100???00020000000000?00000??000??000?0000?1000??????0010000000?1001000?01?0000021100000010101100010011000?0000011001000???000001000000100000000100000?000?00010???0?1000?000000001000000010000?0010A?

*Allosaurus* 00000100000000000000000010000000200001000000000000001000000011101100011010100110000001000000000100111020200100000121000001000010000000001000000000000100000000010000000-000000000000100011001000001000010200111011000000110A

*Abelisaurus* 1111120?1?011??101111112101111101110?000101?11111?1001?111?10???10011??1???1??1?????????101?????????????????????????????????????????????????????????????????????????????????????????????????????????????????????????????????

*Aucasaurus* 11?112??1?0?11???1??11?2??????0????????????110???1????????????????????????????????????0???????????????????1??????????1????????????????1?21111?1?????010?211111111112111001?1??101?11???111???1?0??1?1111??1?1111??0??1111?0B

*Berberosaurus* ???????????????????????????????????????????????????????????????????????????????????????????????1??????220?000??00???0?1?????????1?????????????0????????????????????0??11??????????????????????0101???10110???????????????Car

*Carnotaurus* 111112111?011110110111121011111101111011101110111111111110111???100111?111?111111111110?10100012?1111122111101110121010001111111111111110211?1?10111101002111101111121110011111101111111111?11110?1111????11????1?????????1?

CCG20011 ??????????????????????????????????????????????????????????????????????????????????????????????????????110?0111?0?1??211010?0?0000???????1?0????????????????????????????????????????????????????????????????????????????????C

*Ceratosaurus* 0000120010000000010000000001010020100000000000000000000000111?110000111011100111101001000011111210111122100000001210000010111100110?1?010010020000010000011010100000100111011101??00000?1111110101101101101111100010???00Dah

*Dahalokely* ??????????????????????????????????????????????????????????????????????????????????????????????1???????22111101010121110001111111???????????????????????????????????????????????????????????????????????????????????????????D

*Deltadromeus* ???????????????????????????????????????????????????????????????????????????????????????????????????????????????????????????????????????0??????1????1??111111?10?0?????????????????????????????1?0?01100?1121?111?1100?0????0

*Ekrixinatosaurus* 1??1?011??01110?????1110?11?1?1?0????1???11???????????1????1??????????????????????11??01?0????1???????222?11?001?1???11???????1?????0?0???111??0????????????????????????????1?????1????111?????0?011?1110??01??1?00????1???E

*Elaphrosaurus* ??????????????????????????????????????????????????????????????????????????????????????????????1??????11000111001100211010111100011111?00101??1?1??1?0111111111010??10?01????1011011000011?0?0100?1101001121010010010?????00E

*Eoabelisaurus* ?0????????????????000100000010020101001000?10?01??0??01?011?????00?1??????????????????00???0?1??????221?110?0101?1010001011111011111?1021110200??110?0000??101010110111111111011?000?11?-11???0101101101111??000111?00Genusa

*Genusaurus* ???????????????????????????????????????????????????????????????????????????????????????????????1???????2??????0???????????0???1???1??????????????????????????????????????????11?01??1111????????0?0201???11????????????????G

*Genyodectes* 0???0????????????????????????????????????????????????????????????????????????????000??0001111???????????????????????????????????????????????????????????????????????????????????????????????????????????????????????????????

*Ilokelesia* ?1????????????????????????????11101111010?1???????????????????1???????????????????????????????1???????222110010101210110????1111?????????2??112?011???????????????????????????????????????????????????????????????????111???

*Indosaurus* ??????????????????11?1111?111??????????????????????????????11?????1?????????????????????????????????????????????????????????????????????????????????????????????????????????????????????????????????????????????????????????

*Kryptops* 1????20111011?????????????????????????????????????????????????????????????????????????01100?????????????????????????????????????????????????????????????????????????????????????????????????????????????????????????????????

*Laevisuchus* ??????????????????????????????????????????????????????????????????????????????????????????????1??????????01?1?0011?011?00??????????????????????0????????????????????????????????????????????????????????????????????????????

*Limusaurus* 001?0?0???0?00000?00?000000??000?01???00?0?0?0000??????00?????????????????1-?????0?0-1???????1??0?000?2000?11???10?21??0??11????????????10?00?010011011101??100101?11?00111001110010000?00?0????1?00???1??1????10?1???00Maju

*Majungasaurus* 1111120111011101011111111011111101100010001111011110111111111111101111111121111111001101101000121111112211110101012101000101111111111111210002101101010021111111?112111001????1011111011?11??11?11??0?1011?1111110011111?0Ma

*Masiakasaurus* 00?1?00?111?0?????00000000????00001000100?0?0010?????????????11?1001???????10?1110000110011?0?12?011112210111100111021000111110110111101111001?0111?0111111111010????????11011111?11010??1110110111210?11111111111111110110N

*Noasaurus* 0??1?00111110?0???????????????????????????????????????????0???????????????????????????10011??01?????????01?1?101110??00????????????????????????000?????????????????????????0???????????????????0???????????????????1???????C

Composite_Vesp ????????????????????000000????????????????????????????????????????????????????????????????????11?011?12210111001100210001?1110??101????????0?1????1?011?00111001?10????02?10??111???010???1?0?10??????????????????11111111Ve

*Vespersaurus*_hol ??????????????????????????????????????????????????????????????????????????????????????????????????????????????????????????????0???01??????????1????????????????????????????????111???010???1?0?10??????????????????11111111R

*Rahiolisaurus* 1????????????????????????????????????????????????????????????????????????????????????0??001??????111110001?0011?12?0?00?????????111??????????????????11?????????????????????1??01111010?1?1?10?0?1?11011111?1?11?0010?????0R

*Rajasaurus* 11????????????????1??1111?111????????????????????1?????????1111??01???????211????1?0110?101???12????????????????????????011?111??110?11?0???????0????????????????????????????0???0???110???1???11??111??1?11?1?????0?0??????

*Rugops* 111110??1?0111011101000011110??????????????11110011??????????????????????????????????101101?0???????????????????????????????????????????????????0???????????????????????????????????????????????????????????????????????????

*Skorpiovenator* 11?112111?01111????????01????11111110111011011111??11???1??????????????????1??????111?01???0?0???????????1???010?21?11??????????????????2?111?1?11???????????????????????????1?0110011011?????????11??????????????0?1??1?10M

MNNtig6 ??????????????????????????????????????????????????????????????????????????????????????????????11??????22001010010112011010111100???111?????????000?????????????????????????????????????????????????????????????????????????S

*Spinostropheus*_type ???????????????????????????????????????????????????????????????????????????????????????????????1??????201?011??01??1?00?????????????????????????????????0000???????????????????????????????????0?????0010????????????0?0???V

*Velocisaurus* ????????????????????????????????????????????????????????????????????????????????????????????????????????????????????????????????????????????????????????????????????????????????????????????????0?11??0011?1?111111111??1??1

USNM6415 ?????????????????????????????????????????????????????????????????????????????????????????????????????????????????????????????????????????????????????????10001001???????????????????????????????????????????????????????????

*Afromimus* ???????????????????????????????????????????????????????????????????????????????????????????????????????????????????????????????????????????0001?????????????????????????????????????????????????????????102111??11????1?1???

*Spectrovenator* 11???200??01100111010000110001111100000000011101101111111??11010?1111111?1111?1?000?0100?0?0??1?????2??????10?????????01??1?1?????1???????11???11??????????????????????????11?0111110011???1?10?01??1110?101111110?111??0Ph?

*Berthasaura* 000?010??1110???010?0?000?010??????????????10000??????0?101?01???????1??10?1?010?000?-?-???---1???????2210?1?1?111?011100?111?01??00??1?01110020?1?110110011?10110102?????????1?01111??011?1?1?10?11010???21??????????0???1?

;

END;

**References**

1. Wang, S. *et al.* Extreme ontogenetic changes in a ceratosaurian theropod. *Curr. Biol.* **27**, 144–148 (2017).

2. O’Connor, P. M. The postcranial axial skeleton of *Majungasaurus crenatissimus* (Theropoda: Abelisauridae) from the Late Cretaceous of Madagascar. *J. vertebr* **27**, 127–163 (2010).

3. Rauhut, O. W. M. & Carrano, M. T. The theropod dinosaur *Elaphrosaurus bambergi* Janensch, 1920, from the Late Jurassic of Tendaguru, Tanzania. *Zool. J. Linn. Soc.* **178**, 546–610 (2016).

4. Langer, M. C. *et al.* A new desert-dwelling dinosaur (Theropoda, Noasaurinae) from the Cretaceous of south Brazil. *Sci. Rep.* **9**, 1–31 (2019).

5. Maddison, W. P. & Maddison, D. Mesquite 2. *Manual* 1–258 (2010).

6. Sereno, P. C., Wilson, J. A. & Conrad, J. L. New dinosaurs link southern landmasses in the Mid-Cretaceous. *Proc. R. Soc. B Biol. Sci.* **271**, 1325–1330 (2004).

7. Carrano, M. T., Benson, R. B. J. & Sampson, S. D. The phylogeny of Tetanurae. *J. Syst. Paleontol.* **10**, 211–300 (2012).

8. Carrano, M. T. & Sampson, S. D. The phylogeny of Ceratosauria (Dinosauria: Theropoda). *J. Syst. Palaeontol.* **6**, 183–236 (2008).

9. Sereno, P. C. *et al.* Predatory dinosaurs from the Sahara and Late Cretaceous faunal differentiation. *Science* **272**, 986–991 (1996).

10. Apesteguía, S., Smith, N. D., Valieri, R. J. & Makovicky, P. J. An unusual new theropod with a didactyl manus from the upper cretaceous of patagonia, Argentina. *PLoS One* **11**, 1–41 (2016).

11. ZAHER, H., POL, D., NAVARRO, B. A., DELCOURT, R. & CARVALHO, A. B. An Early Cretaceous theropod dinosaur from Brazil sheds light on the cranial evolution of the Abelisauridae. *Comptes Rendus Palevol* **2020**, (2020).

12. Cerroni, M. A., Agnolin, F. L., Brissón Egli, F. & Novas, F. E. The phylogenetic position of *Afromimus tenerensis* Sereno, 2017 and its paleobiogeographical implications. *J. African Earth Sci.* **159**, 103572 (2019).

13. Samathi, A., Sander, P. M. & Chanthasit, P. A spinosaurid from Thailand (Sao Khua Formation, Early Cretaceous) and a reassessment of *Camarillasaurus cirugedae* from the Early Cretaceous of Spain. *Hist. Biol.* **00**, 1–15 (2021).

14. Filippi, L. S., Méndez, A. H., Juárez Valieri, R. D. & Garrido, A. C. A new brachyrostran with hypertrophied axial structures reveals an unexpected radiation of latest Cretaceous abelisaurids. *Cretac. Res.* **61**, 209–219 (2016).

15. Farke, A. A. & Sertich, J. J. W. An Abelisauroid Theropod Dinosaur from the Turonian of Madagascar. *PLoS One* **8**, (2013).

16. Bonaparte, J. F. . & Novas, F. E. *Abelisaurus comahuensis*, Ng, N. Sp., Carnosauria From the Late Cretaceous of Patagonia. *Ameghiniana* **21**, 259–265 (1985).

17. Canale, J. I., Scanferla, C. A., Agnolin, F. L. & Novas, F. E. New carnivorous dinosaur from the Late Cretaceous of NW Patagonia and the evolution of abelisaurid theropods. *Naturwissenschaften* **96**, 409–414 (2009).

18. Rauhut, O. W. M. The interrelationships and evolution of basal theropod dinosaurs. *Spec. Pap. Paleontol.* **69**, 1–213 (2003).

19. Pol, D. & Rauhut, O. W. M. A middle Jurassic abelisaurid from Patagonia and the early diversification of theropod dinosaurs. *Proc. R. Soc. B Biol. Sci.* **279**, 3170–3175 (2012).

20. Smith, J. B. Dental morphology and variation in *Majungasaurus crenatissimus* (theropoda: Abelisauridae) from the late cretaceous of madagascar. *J. Vertebr. Paleontol.* **27**, 103–126 (2007).

21. Rauhut, O. W. M. Braincase structure of the Middle Jurassic theropod dinosaur *Piatnitzkysaurus*. *Can. J. Earth Sci.* **41**, 1109–1122 (2004).

22. Rauhut, O. W. M. & Rauhut, O. W. M. Provenance and anatomy of *Genyodectes serus*, a large-toothed ceratosaur (Dinosauria: Theropoda) from Patagonia. *J. Vertebr. Paleontol.* **24**, 894–902 (2004).

23. Carrano, M. T., Sampson, S. D. & Forster, C. A. The osteology of *Masiakasaurus knopfleri*, a small abelisauroid (Dinosauria: Theropoda) from the Late Cretaceous of Madagascar. *J. Vertebr. Paleontol.* **22**, 510–534 (2002).

24. Novas, F., Ezcurra, M. & Agnolin, F. Humerus of a basal abelisauroid theropod from the Late Cretaceous of Patagonia. *Rev. del Mus. Argentino Ciencias Nat.* **8**, 63–68 (2006).

25. Bonaparte, J. F. The gondwanian theropod families Abelisauridae and Noasauridae. *Hist. Biol.* **5**, 1–25 (1991).

26. Novas, Dalla Vecchia, F. & Pais, D. Theropod pedal unguals from the Late Cretaceous (Cenomanian) of Morocco, Africa. *Rev. del Mus. Argentino Ciencias Nat.* **7**, 167–175 (2005).
